# Hox gene expression during development of the phoronid *Phoronopsis harmeri*

**DOI:** 10.1186/s13227-020-0148-z

**Published:** 2020-02-10

**Authors:** Ludwik Gąsiorowski, Andreas Hejnol

**Affiliations:** 1 Sars International Centre for Marine Molecular Biology, University of Bergen, Thormøhlensgate 55, 5006 Bergen, Norway; 2 Department of Biological Sciences, University of Bergen, Thormøhlensgate 55, 5006 Bergen, Norway

**Keywords:** Lophophorata, Spiralia, Biphasic life cycle, Intercalation, Life history evolution, Body plan, Indirect development, Lox2, Head, Brain

## Abstract

**Background:**

Phoronida is a small group of marine worm-like suspension feeders, which together with brachiopods and bryozoans form the clade Lophophorata. Although their development is well studied on the morphological level, data regarding gene expression during this process are scarce and restricted to the analysis of relatively few transcription factors. Here, we present a description of the expression patterns of Hox genes during the embryonic and larval development of the phoronid *Phoronopsis harmeri*.

**Results:**

We identified sequences of eight Hox genes in the transcriptome of *Ph. harmeri* and determined their expression pattern during embryonic and larval development using whole mount in situ hybridization. We found that none of the Hox genes is expressed during embryonic development. Instead their expression is initiated in the later developmental stages, when the larval body is already formed. In the investigated initial larval stages the Hox genes are expressed in the non-collinear manner in the posterior body of the larvae: in the telotroch and the structures that represent rudiments of the adult worm. Additionally, we found that certain head-specific transcription factors are expressed in the oral hood, apical organ, preoral coelom, digestive system and developing larval tentacles, anterior to the Hox-expressing territories.

**Conclusions:**

The lack of Hox gene expression during early development of *Ph. harmeri* indicates that the larval body develops without positional information from the Hox patterning system. Such phenomenon might be a consequence of the evolutionary intercalation of the larval form into an ancestral life cycle of phoronids. The observed Hox gene expression can also be a consequence of the actinotrocha representing a “head larva”, which is composed of the most anterior body region that is devoid of Hox gene expression. Such interpretation is further supported by the expression of head-specific transcription factors. This implies that the Hox patterning system is used for the positional information of the trunk rudiments and is, therefore, delayed to the later larval stages. We propose that a new body form was intercalated to the phoronid life cycle by precocious development of the anterior structures or by delayed development of the trunk rudiment in the ancestral phoronid larva.

## Background

Hox genes encode a family of transcription factors present in Bilateria and Cnidaria [1–4], which bind with their conserved homeodomain directly to regulatory regions of downstream genes and activate or suppress their expression (e.g. [5–7]). In many clades, Hox genes are differentially expressed in the early developmental stages along the anterior–posterior axis of the developing embryo, being one of the important components of molecular patterning of axial identities [4–6, 8–10]. The diversity of Hox genes present in extant Bilateria originated likely by multiple duplication events, which resulted in the physical linkage of Hox genes in the genomes of many Bilateria, the so-called Hox clusters (e.g. [9, 11, 12]. It is possible to discriminate organized, split and disorganized Hox clusters, depending on the level of their organization [7, 12] and in certain Bilateria the Hox genes are expressed in roughly the same order as they are located in the cluster: a phenomenon referred to as collinearity [6, 9, 11]. The correspondence between position of the gene in the cluster and onset of its expression might have a temporal (during development) or spatial (along body axis) character and accordingly it is possible to discriminate between the temporal and spatial collinearity. It has been proposed that collinearity, especially the temporal one, is a major factor responsible for conservation (or maybe even formation) of the ordered Hox cluster in the genome [9, 11–16].

**Fig. 1:**
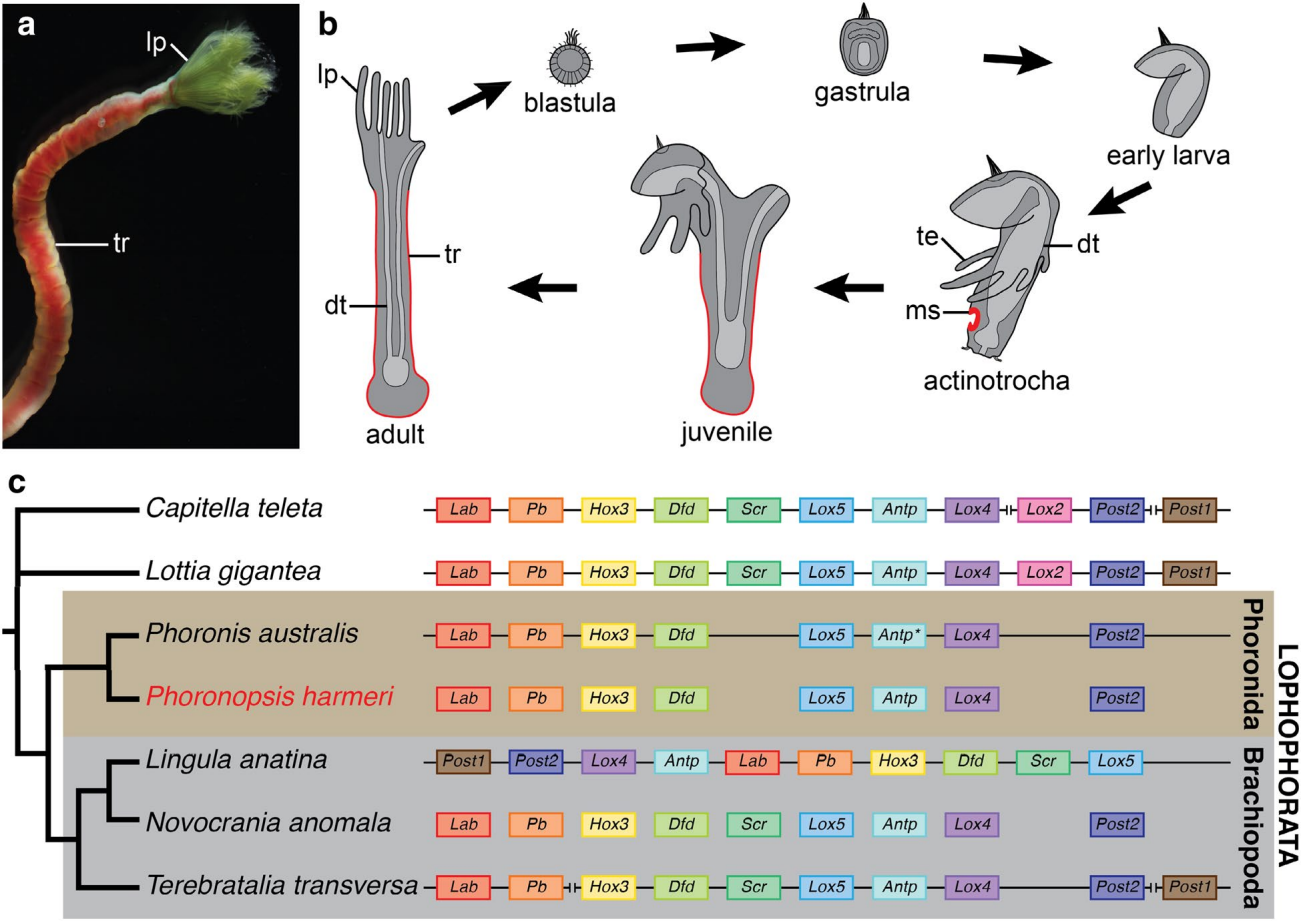
*Phoronopsis harmeri*: morphology of the anterior part of living animal (**a**) and scheme of its life cycle (**b**). Hox clusters organization and Hox genes complement in various Spiralia (**c**), based on [16, 89, 107]. Metasomal sac and adult trunk originating from it are marked in red in **b**. Gene *antp* from *Phoronis australis* (marked with asterisk) was originally described as *lox2* (see text for discussion). For *Phoronopsis harmeri* and *Novocrania anomala* only the Hox complement is available (data on cluster organization are missing). The vertical bars in C indicate boundaries of the particular scaffolds of the split Hox clusters. Abbreviations: *dt* digestive tract, *lp* lophophore, *ms* metasomal sac, *te* larval tentacles, *tr* adult trunk

Although expression of Hox genes has been described during embryonic and larval development of many animals representing diverse evolutionary lineages [4, 16–49], there are still some clades for which information about Hox expression during development is lacking. Among them are phoronids, marine, sessile worms, which feed using a specialized filter apparatus, the so-called lophophore (*lp* in Fig. 1a). Due to the presence of lophophore, Phoronida have been traditionally united with two other clades—Ectoprocta (Bryozoa) and Brachiopoda—into the group called Lophophorata [50, 51], which recently gained support as a valid clade from several transcriptomic and phylogenomic studies [52–55]. Although originally the Lophophorata were considered as deuterostomes [50, 51], molecular data showed their protostome affinity [56] and currently the lophophorates occupy a well-supported position within the clade of Spiralia [52–55, 57]. Most phoronids develop through a distinctive planktotrophic larval stage, called actinotrocha [58–61]. After a prolonged planktonic life, the actinotrocha larva settles and undergoes drastic metamorphosis (Fig. 1b), during which the rudiment of the body wall of the adult worm, the so-called metasomal sac (ms, Fig. 1b), is everted and the rudiments of the adult internal organs descent from the larval body to the newly formed juvenile worm (Fig. 1b) [60, 61]. The only exception from this pattern is *Phoronis ovalis*, which is a sister group to the remaining phoronids [62–64] and which develops through the creeping slug-like larva [60]. After a few days of development the active larva of *P. ovalis* settles and acquires a smooth hemispherical shape [60]. Unfortunately, the degree of the metamorphosis-related remodeling of internal structures in *P. ovalis* remains poorly examined.

The phoronid development has been well studied on the morphological level (e.g. [58–61, 65–85]), including preliminary cell lineage, blastomere ablation and fate mapping studies [86–88]. However, information about the molecular patterning is limited to the single study of nine transcription factors (which include anterior, posterior and endomesodermal markers) during the development of *Phoronopsis harmeri* [85]. Importantly, information about expression of Hox genes during development of any phoronid species is still lacking [40, 59].

Recently, Luo et al. have demonstrated that in phoronid *Phoronis australis* a Hox cluster is highly organized with all of the eight phoronid Hox genes forming a single cluster that retains the ancestral spiralian order of genes ([89], also Fig. 1c). This is in contrast to brachiopods, the putative close relatives of Phoronida, where various level of Hox cluster disorganization was shown (Fig. 1c) and temporal and spatial collinearity is missing [16, 40, 89, 90]. Therefore, it remains important to examine whether phoronid Hox genes are also expressed in the spatio-temporally collinear manner during development, which would correspond with the retention of the organized Hox cluster shown in this clade.

Phoronids exhibit a biphasic life cycle with planktotrophic larvae that transform into the juvenile in a catastrophic metamorphosis event (Fig. 1b; e.g. [59, 60, 73, 75, 81, 82]), which is much more drastic than relatively gradual metamorphosis of most Spiralia. Importantly, the A–P axis of the larva is profoundly altered during metamorphosis [60, 77, 81, 82] and results in the U-shaped organization of the internal structures of the juvenile worm (Fig. 1b). In animals with pronounced metamorphosis Hox genes might exhibit noticeable differences in the expression patterns during development of larval and adult bodies. In pilidiophoran nemerteans and indirectly developing hemichordates it has been demonstrated that Hox genes are involved in patterning of only adult bodies [37, 38], while in tunicates and sea urchins different sets of Hox genes are expressed during larval and adult body development [21, 22, 44, 47]. On the other hand, in animals with non-catastrophic metamorphosis (e.g. cephalochordates, mollusks, annelids or brachiopods), the Hox genes seem to pattern both the larval and adult body plans in a relatively similar way [31, 39, 40, 46, 48]. However, studies focusing on metamorphosis-related differences of Hox gene expression in Bilateria are still limited to a relatively few evolutionary lineages [40, 91]. Therefore, the comparison of Hox gene expression between the embryonic and larval development and the development of the metasomal sac in phoronids might shed new light into the understanding of the evolution of differential genetic control of the axis patterning in animals with extreme metamorphosis.

In this study, we investigated the Hox genes complement and their expression patterns during the development of the phoronid *Phoronopsis harmeri,* for which the extensive data on the morphological aspects of the development and some molecular data on the A–P axis are available [66, 72, 75–78, 80–82, 84, 85]). Our aim was to answer whether phoronid Hox genes show staggered expression along the A–P axis at any of the developmental stages as well as to examine if there are traces of temporal collinearity that could hint to the presence of a Hox cluster as described for another phoronid *P. australis* [89]. We also wanted to investigate whether there are differences in the Hox gene expression (and possibly in the patterning of the A–P axes) between the larva and the rudiment of the forming juvenile worm and compare our findings with other species that exhibit extreme metamorphosis.

## Results

### Hox complement and gene orthology

**Fig. 2:**
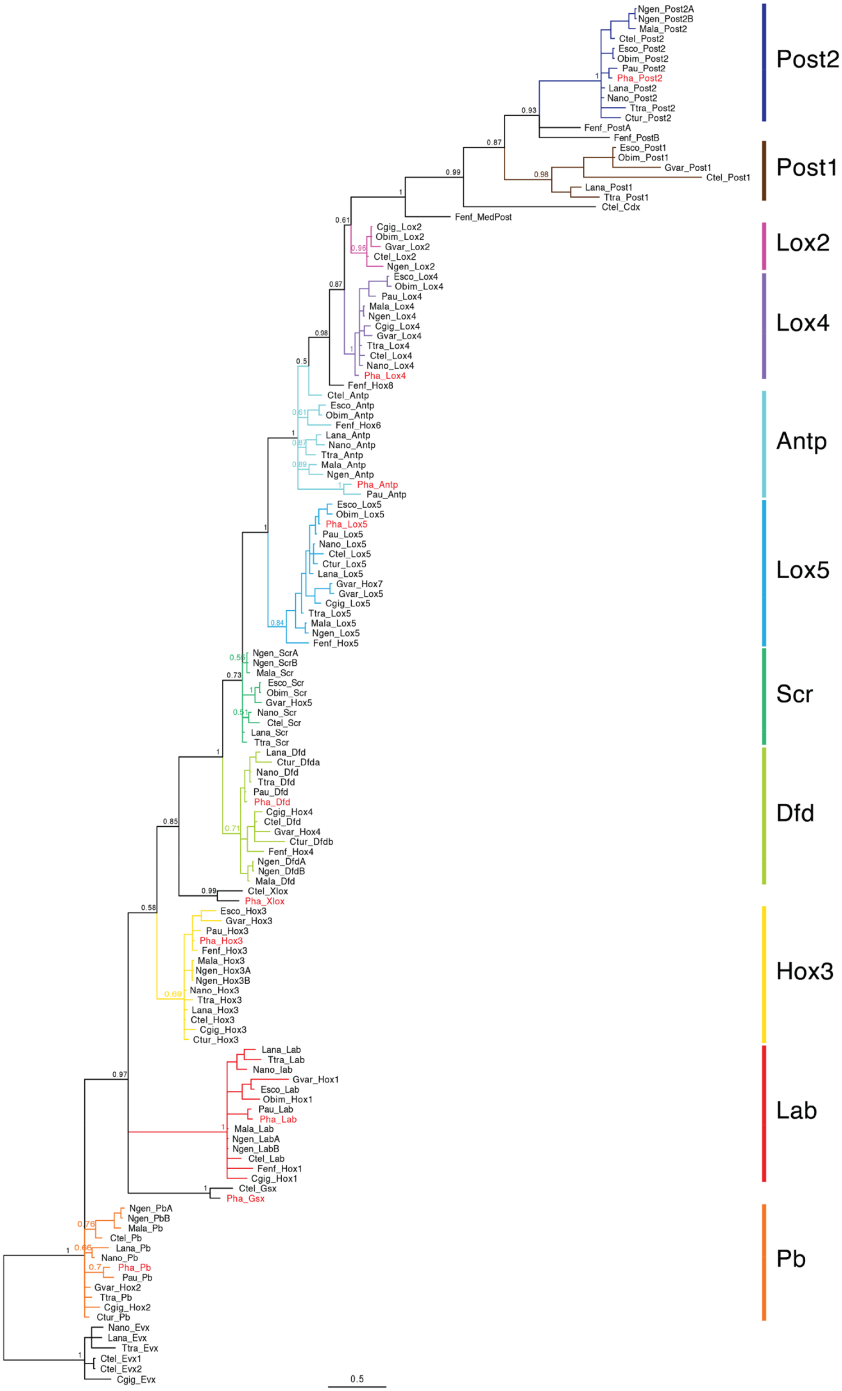
Bayesian phylogeny under JTT + I + G substitution model of the amino acid sequences of spiralian Hox genes homeodomains, including phoronid sequences. Genes from *Ph. harmeri* are marked in red. Posterior probability values are shown for important clades. Full species names and sequences accession number are provided in Additional file 1: Table S1

We identified eight Hox genes in the transcriptome of *Ph. harmeri* and our phylogenetic analysis allowed their assignment to particular orthology groups (Fig. 2). Those genes represent orthologues of the genes *labial* (*lab*), *proboscipedia* (*pb*), *hox3*, *deformed* (*dfd*), *lox5*, *antennapedia* (*antp*), *lox4* and *post2* (Figs. 1c and 2). Moreover, in addition to the paraHox gene *cdx* reported by Andrikou et al. [85], we identified two other paraHox genes in the transcriptome of *Ph. harmeri*—*gsx* and *xlox*. Most of the Hox orthologues form distinct clades in our phylogenetic tree (Fig. 2). Sequences from the three orthologues (*pb*, sex combs reduced (*scr*) and *antp*) do not form clades but rather grades of similar sequences (Fig. 2), which nevertheless allow the exact orthology assessment. We found that the gene identified by Luo et al. as *lox2* in the genome of *P. australis* [89] and its orthologue in *Ph. harmeri* do not fall into the clade containing *lox2* sequences from other Spiralia, but instead they group in the grade containing *antp* sequences. Accordingly, sequence of those two phoronid genes lack most of the residues proposed as signature of *lox2* by de Rosa et al. (Additional file 1: Fig. S1; [92]).

### Embryonic and larval development of ***Ph. harmeri***

**Fig. 3:**
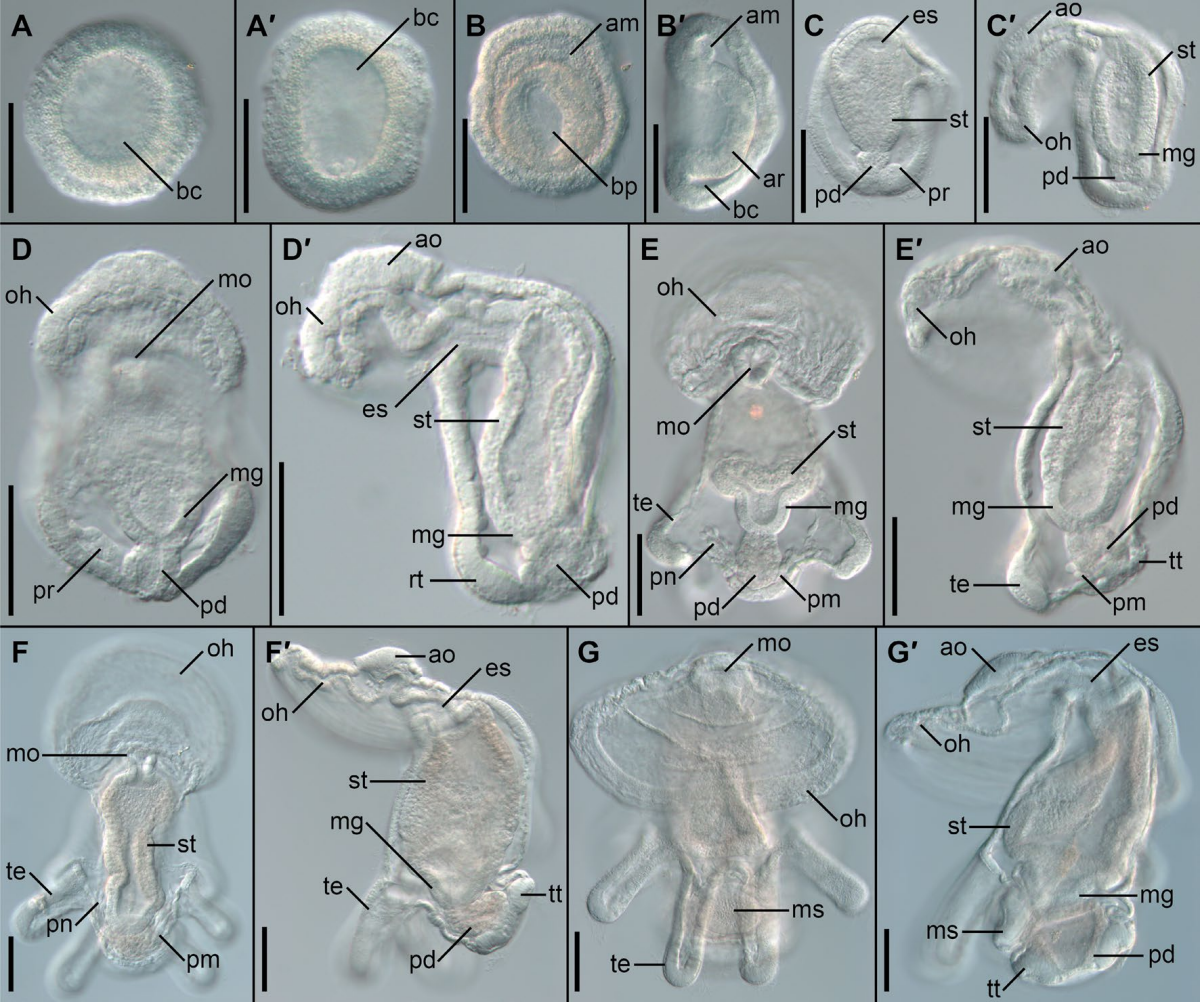
Development of *Phoronopsis harmeri*. Blastula, 12 hpf (**A**, **A’**); gastrula, 24 hpf (**B**, **B’**); early larva, 42 hpf (**C**, **C’**); pre-tentacle larva, 56 hpf (**D**, **D’**); actinotrochae: 3 dpf (**E**, **E’**), 5 dpf (**F**, **F’**) and 7 dpf (**G**, **G’**). For each developmental stage the left panel shows embryo or larvae in dorso-ventral view and right panel (marked as’) in lateral view with ventral to the left; anterior is to the top on all panels. Scalebars 50 μm. *am* anterior mesoderm, *ao* apical organ, *ar* archenteron wall, *bc* blastocoel, *bp* blastopore, *es* esophagus, *mg* midgut, *mo* mouth opening, *ms* metasomal sac, *oh* oral hood, *pd* proctodeum, *pm* posterior mesoderm, *pn* protonephridium, *pr* protonephridial rudiment, *rt* tentacle rudiment, *st* stomach, *te* tentacle, *tt* telotroch

Embryos and larvae of *Ph. harmeri* are relatively transparent and many aspects of their morphology can be easily observed with the light microscope using Nomarski interference contrast (Fig. 3). At 9 °C the blastula stage is reached at about 6–8 h post-fertilization (hpf). Around 12 hpf a swimming blastula with a large blastocoel (*bc*) is formed (Fig. 3A, A’). At 20 hpf the gastrulation process is initiated, which leads to the formation of the gastrula (Fig. 3B, B’) that displays a distinctive blastopore (*bp*), the archenteron (*ar*) and the anterior mesoderm (*am*). Subsequently, the embryo (including the archenteron) elongates along the A–P axis and the oral hood (*oh*) develops anteriorly leading to the formation of the early larval stage, at approximately 40 hpf (Fig. 3C, C’). In the posterior part of the early larva the proctodeum (*pd*) develops, which merges with the posterior midgut (*mg*), forming a larval digestive system. Ventrally to the proctodeum the first undifferentiated rudiment of the protonephridia is present (*pr* in Fig. 3C, C’). At 60 hpf the pre-tentacle larval stage is reached (Fig. 3D, D’), which possesses a through-gut (with esophagus, *es*; stomach, *st*; midgut, *mg*; and proctodeum, *pd*), an apical organ (*ao*), protonephridial rudiments (*pr*) and rudiments of the first three pairs of tentacles (*rt*). Three days post-fertilization (dpf) larvae can be already identified as early 6-tentacle actinotrocha (Fig. 3E, E’) due to the presence of three pairs of well-defined tentacles (*te*). At this stage the larval protonephridia reach their definite branching form (*pn*, Fig. 3E), the rudiments of posterior mesoderm are morphologically distinguishable (*pm,* Fig. 3E) and the posterior telotroch starts to form around the anal opening (*tt*, Fig. 3E’). At 5 dpf (Fig. 3F, F’) the telotroch is fully formed, while the posterior mesoderm forms rudiments of the posterior coelom compartment (metacoel). The actinotrocha reach the 8-tentacle stage at 7 dpf (Fig. 3G, G’). At this stage the post-tentacular region of the body (larval trunk) elongates and the metasomal sac, a rudiment of the body wall of the prospective adult worm, is formed (*ms*, Fig. 3G, G’). The metasomal sac at this stage appears as an ectodermal thickening located on the ventral side under tentacle bases.

The actinotrocha of *P. harmeri* develops further during a prolonged planktonic life (2 weeks up to few months). During this process subsequent pairs of tentacles are added on the dorsolateral sides, while metasomal sac extends, forming elongated structure on the ventral side of the larvae [76, 81, 82]. The actinotrocha of *P. harmeri* reaches metamorphosis competence at the 24-tentacle stage [76, 82]. The morphological details of the embryonic and larval development of *Ph. harmeri* are well described elsewhere [66, 72, 75–78, 80–82, 84, 85], therefore we did not examined further the embryonic and larval morphology.

### Hox gene expression

We did not detect expression of any of the Hox genes in blastula and gastrula stages (Additional file 1: Fig. S2), despite the fact that the expression of other genes, used as positive control, can be easily detected on those developmental stages (and was also reported elsewhere [85]). Additionally, the signal from the probes develops usually faster in embryos compared to larval stages. As we detected signal from all of our molecular probes on later larval stages (see below) we conclude that none of the Hox genes is expressed to a detectable degree before 42 hpf.

**Fig. 4:**
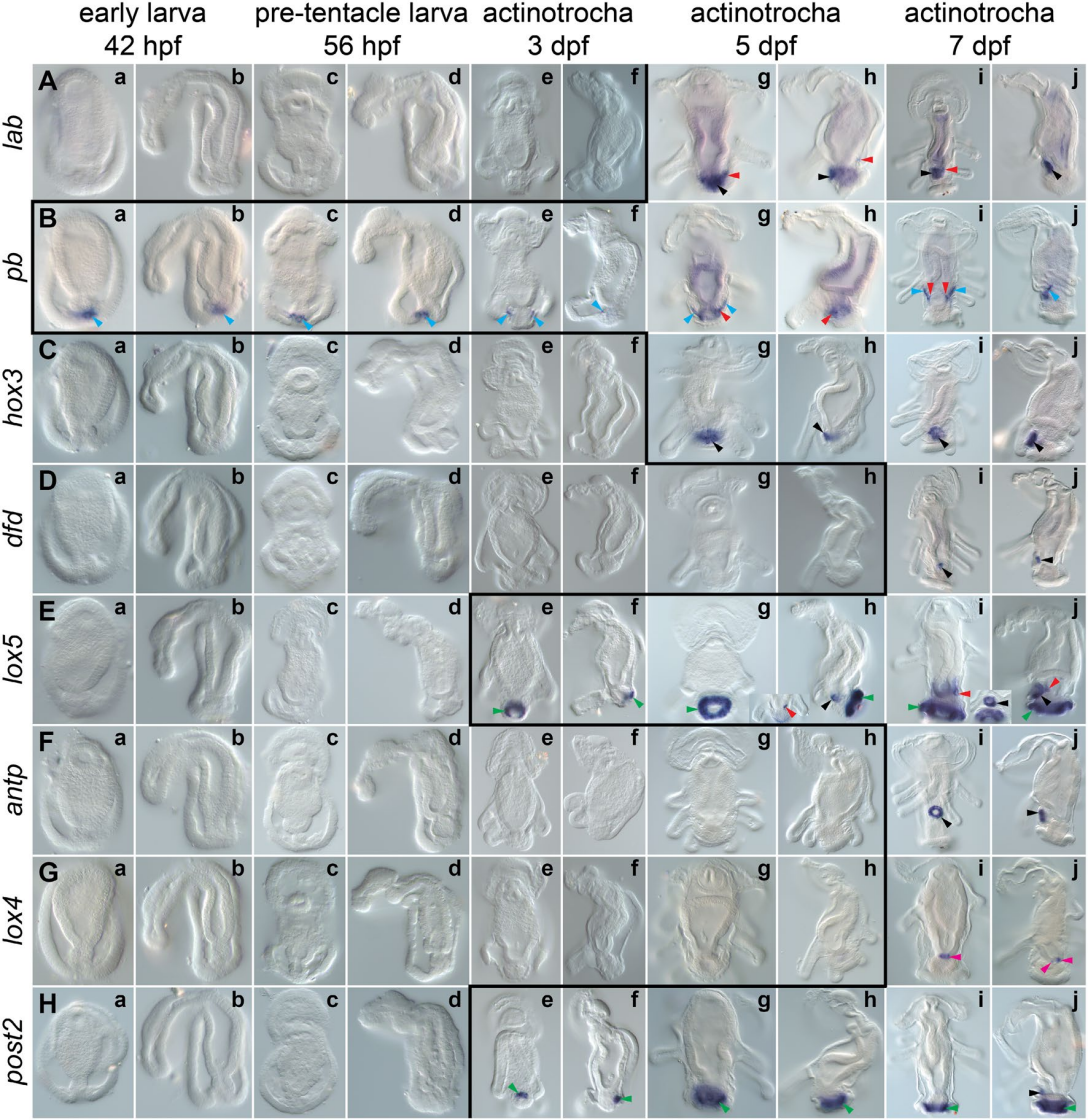
Whole-mount in situ hybridization of each Hox gene during larval development of *Phoronopsis harmeri*. Name of each hybridized gene is shown on the left, while developmental stages are indicated on the top. All the stages are presented with anterior to the top. Larvae on panels *a*, *c*, *e*, *g* and *i* are in dorso-ventral view, whereas larvae on panels *b, d*, *f*, *h* and *j* in lateral view with ventral to the left. The black line indicates the onset of expression of each Hox gene based on in situ hybridization data. Black arrowheads indicate expression in the metasomal sac, blue arrowheads expression in the protonephridia, red arrowheads expression in the mesoderm, green arrowheads expression in the telotroch and magenta arrowheads expression in the digestive system. The detailed expression patterns are described in the text. Photographs are not to scale

**Fig. 5:**
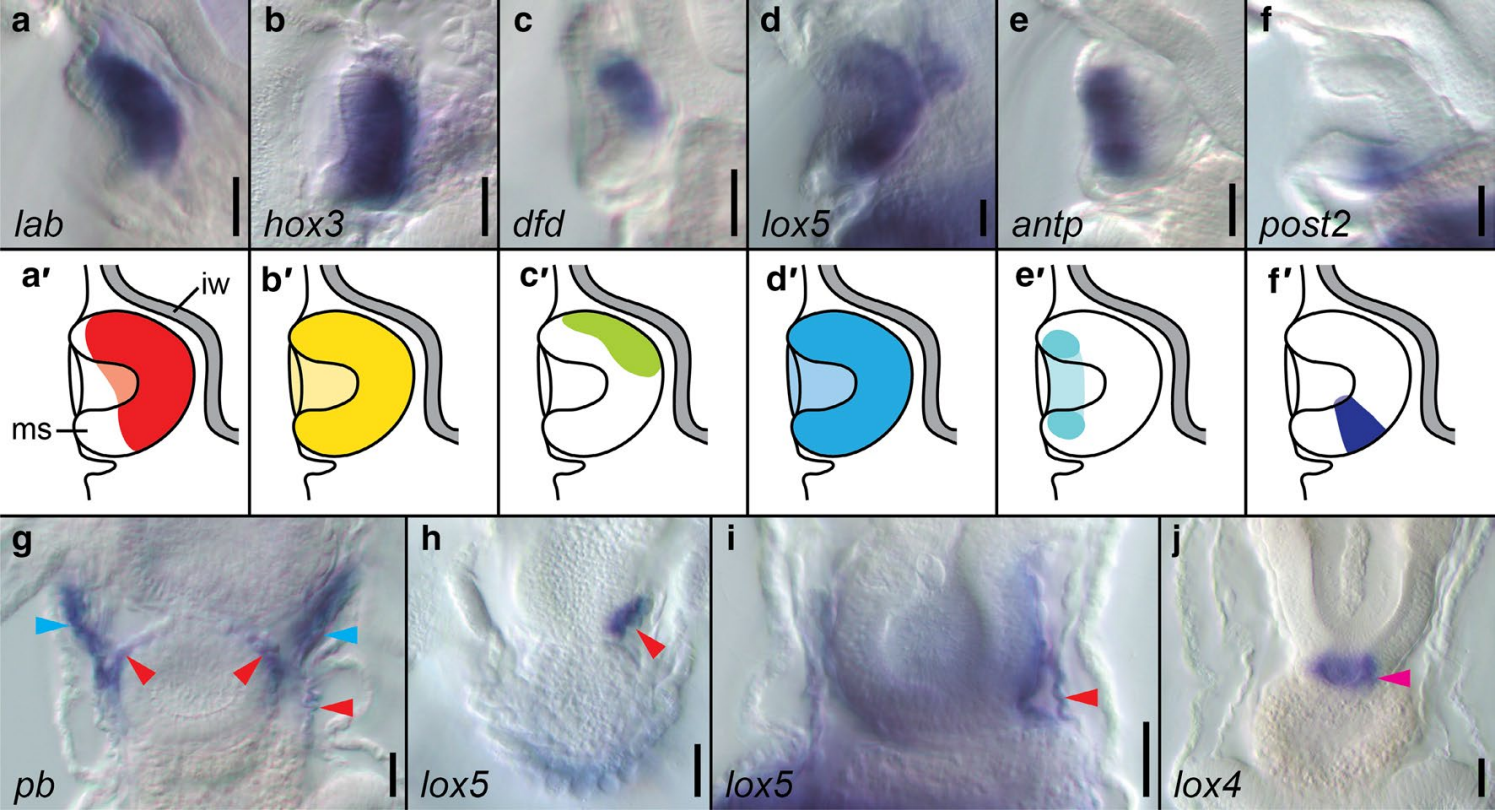
Details of the expression of some of the Hox genes in the actinotrocha larvae of *Phoronopsis harmeri*. Expression of the Hox genes in the metasomal sac of 8-tentacle actinotrochae (**a**–**f**) and schematic interpretation of those expression patterns (**a’**–**f’**). Expression of *pb* in the 8-tentacle actinotrocha (**g**). Expression of *lox5* in the left mesoderm of late 6-tentacle (**h**) and 8-tentacle actinotrocha (**i**). Expression of *lox4* in the digestive system of 8-tentacle actinotrocha (**j**). Scale bars 25 μm. *ms* metasomal sac, *iw* intestinal wall. Blue arrowheads indicate expression in the protonephridia, red arrowheads expression in the mesoderm and magenta arrowhead expression in the digestive system

Expression of the anterior Hox gene *lab* is detected for the first time during development at the late 6-tentacle actinotrocha stage (Fig. 4A g, h). The gene is expressed in the ventro-posterior ectodermal domain, between the tentacle bases and the telotroch (black arrowhead, Fig. 4A g and h) and in the paired domains of the dorso-lateral posterior mesoderm (red arrowheads, Fig. 4A g and h). Both of the expression domains persist to the 8-tentacle actinotrocha stage (Fig. 4A i and j). At this developmental stage the ectodermal domain is part of the metasomal sac, where *lab* is expressed in the cells of the anterior and bottom portion of the sac (Fig. 5a, a’).

The second anterior Hox gene, *pb*, is the earliest expressed among all Hox genes in *Ph. harmeri* as its expression can be already detected in the early larva stage (42 hpf) in some of the cells of the protonephridial rudiment (blue arrowheads, Fig. 4B a, b). This expression domain remains in the pre-tentacle stage (56 hpf, 4B c, d) and early and late 6-tentacle actinotrocha (Fig. 4B e, f). In late 6-tentacle actinotrochae the gene additionally labels a portion of the posterior mesoderm (red arrowheads, Fig. 4B g, h). In 8-tentacle actinotrochae *pb* is expressed in larval protonephridia (blue arrowheads, Figs. 4B i, j; 5g) and in two mesodermal domains, surrounding the metasomal sac (red arrowheads, Figs. 4B i, j; 5g).

*Hox3* expression is detected in the late 6-tentacle actinotrochae in an ectodermal domain between the tentacle bases and telotroch (black arrowhead, Fig. 4C g, h). At the 8-tentacle actinotrocha stage *hox3* is uniformly and exclusively expressed in the ectodermal cells of the metasomal sac (black arrowheads, Figs. 4C i, j; 5b, b’).

*Dfd* expression initiates only at the 8-tentacle actinotrocha stage (Fig. 4D i, j), where the gene is expressed in a small, more proximal portion of the developing metasomal sac (Fig. 5c, c’).

Transcripts of the gene *lox5* are detected first in the early 6-tentacle actinotrocha in posterior cells of the developing telotroch (green arrowhead, Fig. 4E e, f). Later on, *lox5* remains expressed in the telotroch, expanding its expression domain to the entire structure (green arrowheads, Fig. 4E g–j). Two additional expression domains of *lox5* also appear: the metasomal sac rudiment (black arrowhead, Fig. 4E h), which later encompasses the entire metasomal sac (black arrowheads Fig. 4E j and inset between i and j; Fig. 5d, d’), and an asymmetric domain in the left ventro-lateral posterior mesoderm, located between metasomal sac, midgut and left body wall (red arrowheads Fig. 4i, j and inset between g, h; Fig. 5h, i).

Expression of *antp* is not detected until the 8-tentacle actinotrocha stage. Transcripts of the gene are found in ectodermal cells around the opening of the metasomal sac (black arrowheads, Fig. 4F i, j; Fig. 5e, e’), which in a dorso-ventral view look like a ring on the ventral body surface between the tentacles base and the telotroch (Fig. 4F i).

Similarly, *lox4* expression is not detected until the 8-tentacle actinotrocha stage, where the gene exclusively labels the ring of the cells at the junction between midgut and proctodeum (magenta arrowheads, Figs. 4G i, j; 5j).

The only posterior Hox gene, *post2*, is expressed from the early 6-tentacle actinotrocha (3 dpf) in the telotroch (green arrowheads, Fig. 4H e, f), initially in the posterior portion of the organ but later on the expression domain uniformly surrounds the anus (green arrowheads, Fig. 4H g–j). However, compared to *lox5* expression (which also demarcates the telotroch), *post2* labels only the inner ring of epidermal cells of the organ (compare Fig. 4E g–j, H g–j) and not the entire structure. At the 8-tentacle actinotrocha stage the gene *post2* is additionally expressed in the small posterior portion of the metasomal sac (black arrowhead, Figs. 4H j, 5f, f’).

### Head-specific genes

**Fig. 6:**
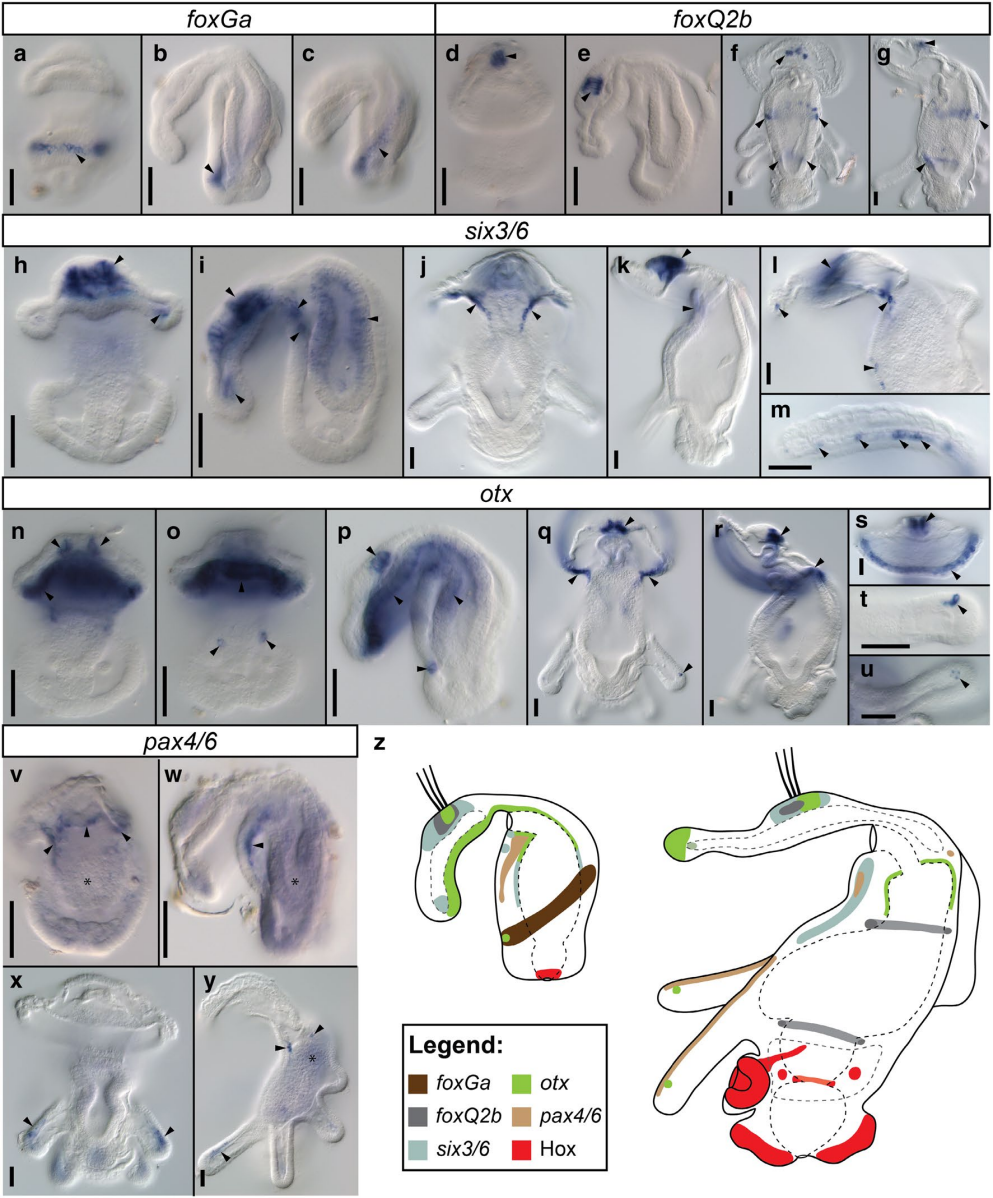
Expression of head-specific genes in early larva (**a**–**e**, **h**, **i**, **n**–**p**, **v**, **w**) and 8-tentacle stage actinotrocha (**f**, **g**, **j**–**m**, **q**–**u**, **x**, **y**) of *Phoronopsis harmeri* and comparison of expression of head-specific and Hox genes in both larval stages (**z**). For each panel the name of hybridized gene is shown in the white box above micrographs. Entire larvae in the dorso–ventral (**a**, **d**, **f**, **h**, **j**, **n**, **o**, **q**, **v**, **x**) and lateral (**b**, **c**, **e**, **g**, **i**, **k**, **p**, **r**, **w**, **y**) views. Details of expression in 8-tentacle stage larvae in oral hood and anterior body region (**l**), hood musculature (**m**), apical organ, preoral coelom and rim of the hood (**s**) and tips of the tentacles (**t**, **u**). Black arrowheads point to the particular expression domains (see text for details), while asterisks indicate unspecific background staining. Scalebars 25 μm

In addition to the investigation of Hox genes we tested expression of several head-specific genes in the early larva (42 hpf) and advanced 8-tentacle actinotrocha. The genes, whose expression we investigated, were *foxG* (also known as brain factor-1 or BF-1), *foxQ2*, *six3/6*, *otx* and *pax4/6*, all commonly considered as head markers [38, 45, 89, 93–97]. One of the two *foxG* paralogues (see “Methods” section for details), whose expression we managed to detect, *foxGa*, is expressed in the early larva in the epidermal cells, from which the tentacles will develop (Fig. 6a–c). In 8-tentacle stage the gene expression was not detected (data not shown). We managed to clone one of two *foxQ2* paralogues, *FoxQ2b*, and detected its expression in the apical organ and the adjacent preoral coelom of both early and 8-tentacle stage larvae (Fig. 6d–g). In the 8-tentacle stage the gene was additionally expressed in two endodermal rings—one in the anterior stomach and another at the border between stomach and midgut (Fig. 6f, g). *six3/6* is expressed in the early larvae in the apical organ, hood mesoderm, preoral coelom, postoral ectoderm and in the stomach (Fig. 6h, i) as previously reported [85]. In 8-tentacle actinotrocha *six3/6* is expressed in the apical organ, hood muscles, preoral coelom and some cells along ventral side of anterior digestive system (Fig. 6j–m). In early larvae *otx* is expressed in the apical organ, ventral preoral ectoderm, anterior portion of the digestive tract and in two spots in the ventro-posterior ectoderm, which lay in the prospective tentacular territory (Fig. 6n–p), following the expression pattern described before [85]. In the 8-tentacle stage, *otx* is expressed in the apical organ, rim of the oral hood, preoral coelom, anterior portion of the digestive tract and in the small spots close to the tips of each tentacle (Fig. 6q–u). *pax4/6* is expressed in the early larvae in the two stripes of cells which extend along ventral side of the larva, from mouth to about half of the body length (Fig. 6v, w) and which correspond with the position to the tentacular neurite bundles [78]. In 8-tentacle stage *pax4/6* expression is detected along frontal side of each larval tentacle (Fig. 6x, y), which also corresponds to the subset of tentacular innervation [78] and in the scattered neurons around the anterior digestive tract (Fig. 6y). In general the head-specific genes are broadly expressed in both developmental stages in the body structures anterior to the Hox-expressing territory (Fig. 6Z).

## Discussion

### Hox gene complement in Phoronida

Similar to the results of the investigation of *P. australis* genome, we identified eight Hox genes in *Ph. harmeri*, which represent single copies of the conserved orthologues of the spiralian Hox genes (Figs. 1c, 2). Luo et al. [89] reported that *P. australis* lacks *scr* and *post1* orthologues and we also did not identify orthologues of those two genes in the transcriptome of *Ph. harmeri*, strengthening the idea they were already absent in the common ancestor of all phoronids.

In their paper Luo et al. [89] suggested that *scr*, which is expressed in the shell forming tissues of brachiopods [16, 40], might be lost in Phoronida due to the evolutionary reduction of the shell in this clade. Such interpretation is in accordance with paleontological data, as a fossil cambrian tommotiid, *Eccentrotheca* sp., which has been proposed as a stem group phoronid [98, 99], possessed a mineralized external tube-shaped skeleton. Recent studies favor a sister group relationship between phoronids and ectoprocts [52–55, 100], the latter of which possess a mineralized external skeleton, similar to brachiopods. However, the Hox gene survey using degenerate polymerase chain reaction primers in the ectoproct *Crisularia* (*Bugula*) *turrita* did not retrieve a *scr* sequence [101], which questions the possible correlation between loss of this gene and the reduction of shell secreting tissues in phoronid lineage. Yet, since it is difficult to recover the full hox complement with degenerate polymerase chain reaction, further studies on bryozoan hox genes, utilizing genomic or transcriptomic data, are needed to ascertain whether *scr* is truly missing.

The gene that was identified as *lox2* by Luo et al. [89] in the genome of *P. australis* (and its orthologue in *Ph. harmeri*) was recovered in our gene orthology analysis as orthologue of *antp* (Fig. 2). Inspection of the phylogenetic tree available in Luo et al. shows that the assessment of the orthology of this gene was tentative, since the gene was actually placed outside of the well-defined clade of *lox2* in their analysis [89]. Identification of this gene as *antp* instead of *lox2* is further supported by its position in the genome of *P. australis*, which corresponds to the *antp* position in the spiralian species with conserved, organized Hox clusters (Fig. 1c). Additionally, alignment of those phoronid genes with *antp* and *lox2* shows that they lack typical signatures of *lox2* [92] and instead are more similar to the *antp* sequence (Additional file 1: Fig. S1). Consequently, both phoronid species lack an orthologue of *lox2*, an absence, which is apparently shared by Phoronida with other Lophophorata [16, 89, 90, 101] as well as with some other Spiralia—i.e. Rotifera [34, 102] and Platyhelminthes [42, 103]. *Lox2* was originally described from leeches [104, 105] and later proposed as an evolutionary innovation of Lophotrochozoa ([92], sensu = Spiralia [106]). However, its orthologues are so far identified only in annelids (e.g. [27, 46, 92, 104, 105, 107, 108]), nemerteans [89], molluscs (e.g. [30, 36, 41, 92, 107, 109–112]) and possibly kamptozoans [113] (however, in the latter the *lox2*-like sequence lacks most of the residues considered as *lox2* signature; Additional file 1: Fig. S1). This indicates that *lox2* evolved only after split of the common ancestor of those clades from remaining Spiralia and does not belong to the ancestral hox complement of all Spiralia [16]. Whether the absence of *lox2* in lophophorates is plesiomorphic or represents an evolutionary reversal depends on the position of Lophophorata within Spiralia, which is still debatable and not fully resolved [52–55, 100].

### Hox genes in Phoronida do not show traces of collinear expression

When assuming the presence of a similar gene order in the Hox cluster of *Ph. harmeri* as in *P. australis* then the former does not show any traces of temporally or spatially collinear expression of Hox genes (Fig. 4). This is in stark contrast to other Spiralia, in which at least some of the Hox genes show staggered expression along A–P axis (e.g. [16, 23, 27, 31, 35–37, 39, 41, 45]). The lack of collinear Hox expression in phoronids is especially intriguing taking into account that *P. australis* has highly organized Hox cluster and collinear expression (especially in its temporal aspect) has been proposed as a main evolutionary factor responsible for conservation of Hox cluster organization [9, 11–16, 49]. Therefore, either another mechanism is responsible for Hox cluster conservation in Phoronida or the two discussed phoronid species vary greatly in the cluster organization and/or Hox gene expression patterns.

Six out of eight identified Hox genes are expressed in the metasomal sac (*pb* and *lox4* being the only two, whose expression was not detected in the structure) and already at the stage of 8-tentacle actinotrocha some of those genes (*lab*, *dfd*, *antp*, *post2*) show differentiated expression in a particular region of the sac (Fig. 5), although without any clear pattern along the future A–P axis. However, it is possible that in the competent larvae (at the 24-tentacle stage, when the metasomal sac is a fully formed, elongated structure [81, 82]), the expression of particular Hox genes is restricted to the different regions of the trunk rudiment and shows some traces of staggered expression along the future A–P axis of the worm body. Hence, the future investigation of Hox expression in competent larvae and freshly metamorphosed juveniles can reveal spatial collinearity obliterated in the early stages of metasomal sac development or eventually confirm a lack of collinear Hox expression throughout entire development of phoronids.

### Germ layer-specific expression of Hox genes in Spiralia

Although Hox genes in Bilateria are predominantly expressed in the ectoderm (including nervous system) and their ectodermal expression is often considered as an ancestral feature [14, 28, 34], in various spiralian species certain Hox genes are also expressed in mesoderm, endoderm and clade-specific structures like chaetal sacs or shell fields (e.g. [16, 23, 24, 27, 29, 31, 35, 36, 39–41, 46]; Table 1). Inclusion of the data on Hox expression in Phoronida gives some new insight into the understanding of the evolution of germ layer-specific Hox expression in Spiralia. *Ph. harmeri*, similar to two investigated brachiopod species [16, 40], seems to lack expression of any of the Hox genes in the nervous system, a peculiarity that might actually represent an apomorphy of Lophophorata (Table 1). Three of the Hox genes—*pb*, *hox3* and *dfd*—were shown to be differentially expressed along the A–P axis in the mesoderm of brachiopod larvae [16]. Out of those three genes, only *pb* (which mesodermal expression is actually lacking in craniiformean *Novocrania anomala* [16]) is expressed mesodermally in *Ph. harmeri,* indicating that cooption of *hox3* and *dfd* into mesoderm patterning occurred after the split of brachiopods and phoronids. Comparison of Hox gene expression across Spiralia (Table 1) allows the observation that *pb* is mesodermally expressed in many species and it is likely that mesodermal expression of *pb* represents an ancestral condition in Lophotrochozoa (sensu stricto [106]). On the other hand, the expression of *lox4* in the digestive system of *Ph. harmeri* is a peculiar and derived feature as this gene is expressed in other Spiralia in ectoderm, nervous system or mesoderm. In general, among investigated Spiralia, the Hox genes are rarely expressed in the digestive system (Table 1).

**Table 1:**
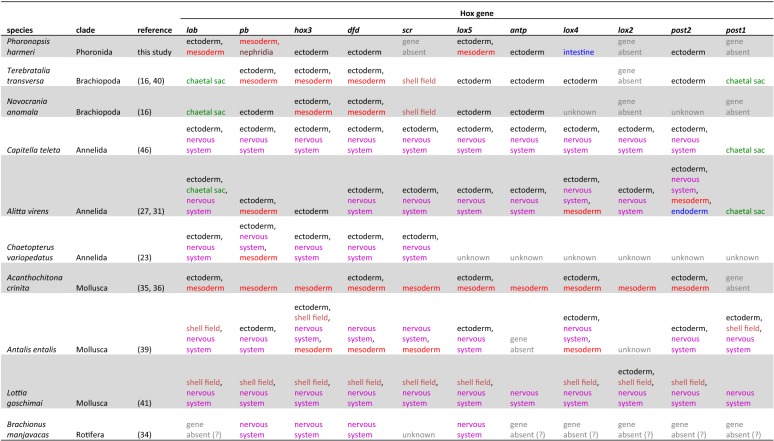
Expression of Hox genes in spiralian species

### Hox gene expression and the nature of actinotrocha larvae

We showed that in *Ph. harmeri* Hox genes are not expressed during embryogenesis, when the larval body is formed, but instead they are expressed mainly in prospective adult structures, namely in the metasomal sac (which will contribute to the adult trunk epidermis), posterior mesoderm (which contributes to the mesodermal structures in the adult trunk), the small posterior portion of the endoderm (which during metamorphosis descent into the trunk rudiment forming the loop of the U-shaped intestine) and the larval telotroch. In most of the investigated Bilateria, Hox genes are already expressed during early developmental stages and, if a biphasic life cycle is present, they are involved in the formation of both larval and adult body plans (e.g. [16, 27, 29–31, 40, 41, 45, 46, 48]). However, there are some animals that, similar to phoronids, deviate from this general pattern. Specifically, in pilidiophoran nemerteans [37] and indirectly developing hemichordates [38], the larvae develop without expressing any of the Hox genes, which instead patterns only the adult body rudiment.

Two evolutionary processes have been proposed to explain these observations. According to the first hypothesis, based on the results from pilidiophoran nemerteans, the new larval form, a pilidium, was intercalated into to the ancestral life cycle of gradually developing nemertean [37, 45]. This intercalation of a larval form caused Hox gene patterning to only be retained during development of the adult worm. In contrast the new larval form, in which the body axis is not aligned with the adult one, uses another molecular mechanism to provide primary positional information to the cells of the developing body [37, 45].

Another concept was proposed to explain the phenomenon observed during larval development of a hemichordate *Schizocardium californicum* [38, 91]. Although metamorphosis in this species is not so drastic [114] and the body axes of both stages are congruent, the larva develops without expression of any Hox genes. Instead, they are expressed only late during larval development and only in the most posterior region of the competent larvae, from which the trunk of the juvenile worm will develop during metamorphosis [38, 114]. Because the larva expresses genes that are usually expressed in the bilaterian head throughout its body, the so-called “head larva”-hypothesis was proposed which states that the larval body represents the homologue of only the head region of the future animal, while the trunk is added later during post-larval development [38]. It has been proposed that ancestrally in Bilateria Hox genes were involved only in the patterning of the trunk, while head developed from the anterior, Hox-free region, the condition, which is still retained in numerous bilaterian lineages [38, 45, 89, 93, 94]. That would explain why tornaria, as a larva composed solely of the head, develops without expression of the Hox genes, which become activated only after the onset of trunk development and pattern only the adult body [38].

Both of those hypotheses (intercalation and “head-larva”) might be applied to explain the Hox expression patterns we observed in *Ph. harmeri*. According to the first hypothesis, the specific actinotrocha larva would represent an evolutionary novelty in the life cycle of phoronids, which was intercalated in the phoronid lineage and that is why it is not patterned by an ancestral Hox gene system. Such an idea is supported by the fact, that the actinotrocha body plan does not bear obvious homology to those of any other spiralian larvae [80, 115–117]. Additionally, similar to the case of pilidium, most of the larval tissues are lost during the drastic metamorphosis event and the larval A–P axis is not aligned with the juvenile one [60, 72, 77, 81, 82]. Moreover, the actinotrocha is lacking in *P. ovalis* [60], which is the sister species to all remaining phoronids [62–64], suggesting that the actinotrocha was not even present in the most recent ancestor of all Phoronida, but instead appeared after the split between *P. ovalis* and the remaining phoronids.

On the other hand, from the morphological point of view, the tentacles of actinotrocha larvae correspond, in case of *Ph. harmeri*, to the tentacles of the lophophore in the adult worm ([73, 82, 116]; Fig. 1b), and the adult lophophore exhibits the molecular signature of a bilaterian head [89]. As tentacles are positioned posteriorly in the early actinotrocha, one can conclude that on a morphological basis the early actinotrocha is mostly composed of the head region. Following such interpretation, all of the Hox genes are expressed in the structures that will contribute to the adult trunk tissues but are not expressed in the developing future head (and hence in the largest portion of the larval body). Accordingly, based on a body region specific transcriptome, it has been demonstrated that in adults of *P. australis* Hox genes are not expressed in the lophophore, while their expression is detectable in the trunk and posterior ampulla [89]. Similarly, in rhynchonelliformean and craniiformean brachiopods none of the Hox genes are expressed in the larval anterior lobe [16, 40], which contributes to the lophophore after metamorphosis [40, 116]. A lack of Hox expression in the adult lophophore tissue (as opposed to the remaining body regions) was also shown for the linguliformean *Lingula anatina*, based on the tissue-specific transcriptomics [89]. Additionally, our study shows that two of the Hox genes (*lox5* and *post2*) are expressed in the telotroch, which represent a truly larval structure, that is lost during metamorphosis [73, 82], therefore Hox genes are indeed, albeit to only a limited degree, involved in larval development. Hox gene expression in the larval telotroch is a result of the telotroch representing a truly “posterior” structure, which belongs to the post-head body region even in the earliest, “head dominated” actinotrocha. The “head larva” interpretation is additionally strengthened by our results of the expression of several head-specific genes in *Ph. harmeri*. Those genes are broadly expressed in the early larvae and 8-tentacle stage, but only in the structures located anteriorly to the Hox-expressing territory (Fig. 6z), resembling conditions in developing tornaria [38].

## Conclusions

Hox gene expression is activated late during the development of *Ph. harmeri*. The larval body develops without expressing any of the Hox genes, which instead are expressed in the tissues of the prospective rudiment of the adult worm and in the telotroch. Such expression might result either from the intercalation of actinotrocha larva into the ancestral life cycle of phoronids or from the fact that the early larva of phoronids represents a “head larva”, which develops without expressing any Hox genes. Our investigation of head-specific genes expression profiles confirms that most of the larval body exhibits head-specific gene expression profile. Those two explanations are not mutually exclusive and we propose that actinotrocha was intercalated into the phoronid life cycle by precocious development of the anterior structures or by delayed development of the trunk rudiment in the ancestral phoronid larva. Such hypotheses can be tested by the investigation of the Hox gene expression during the development of *Phoronis ovalis*, a sister species to all remaining Phoronida, which lacks the actinotrocha larva stage and develops through a creeping, worm-like larva.

## Methods

### Animal collection and fixation

Gravid females of *Ph. harmeri* Pixell, 1912 were collected in Bodega Bay (38° 18′ 51.9012″ N 123° 3′ 12.3012″ W) in California during April and May. Although the California population of *Phoronopsis* is sometimes referred to as separate species *Ph. viridis* [84, 118], we followed the widely accepted interpretation of Joan Rattenbury Marsden, that *Ph. viridis* is in fact a younger synonym of *Ph. harmeri* [119]. The animals were opened in the laboratory and eggs (fertilized during dissection by sperm stored in the coelom of females) were transferred to the clean cultures with filtered see water (as described in, e.g. [78, 84, 85]). Embryos are initially lecithotrophic, but, after formation of the gut, larvae require feeding, hence concentrated *Rhodomonas* or *Rhinomonas* algae were added to the cultures. Water in the larval cultures was exchanged every 2–3 days, followed by the addition of fresh algae. Embryos and larvae on desired developmental stages were relaxed with 8% MgCl_2_, fixed in 3.7% formaldehyde and subsequently washed in phosphate buffer with 0.1% Tween-20. Fixed animals were stored in 100% methanol in − 20 °C.

### Hox genes identification and orthology assessment

We searched the transcriptome of *Ph. harmeri* with reciprocal TBLASTN using eight Hox protein sequences from *Phoronis australis*. The top ten homeodomain-containing BLAST hits from each search were blasted back against the protein database at NCBI (http://blast.ncbi.nlm.nih.gov/) and if any Hox gene was among top reciprocal hits, the sequence was considered to be a putative Hox gene. We identified eight sequences, which passed this reciprocal test and translated them to the protein sequences using CLC Main Workbench 7. Orthology of particular phoronid Hox genes was assessed based on the results of phylogenetic analysis. In order to construct the alignment, amino acid sequences of Hox transcription factors and nucleotide sequences of Hox genes from several spiralian species were obtained from GenBank (https://www.ncbi.nlm.nih.gov/genbank/), the ENSEMBL genome data base (https://www.ensembl.org/index.html) and the website of Marine Genomics Unit of Okinawa Institute of Science and Technology (http://marinegenomics.oist.jp). For the nucleotide sequences, ORFs were determined based on BLAST results at NCBI and sequences were translated into proteins using CLC Main Workbench 7. All spiralian sequences used in this study with their source and accession number are provided in the Additional file 1: Table S1.

The spiralian Hox protein sequences, including putative Hox genes of *Ph. harmeri*, were aligned in CLC Main Workbench 7 and then the alignment was manually trimmed to contain the conserved homeodomain (60 amino acids), five aa 5′ of the homeodomain, and eight aa 3′ of the homeodomain (the trimmed alignment in FASTA format is available in the Additional file 1). Additionally, several spiralian *Evx* sequences were added as an outgroup. ProtTest3 [120] was used to determine the best-fitting substitution model (JTT+I+G). Bayesian analysis was run in MrBayes v3.2.6 [121, 122] with the JTT+I+G substitution model in two independent runs, each with four Markov chains (three heated and one cold) with 3.000.000 generations sampled every 500 generations. The first 25% of samples were discarded as burn-in and the remaining trees were used to calculate posterior probability values and construct the consensus tree, which was visualized and adjusted in FigTree v1.4.3.

All new sequences obtained and identified in this study were uploaded to the GenBank (accession numbers MN443105–MN443114).

### Gene cloning and probe synthesis

Fragments of each Hox gene were amplified from cDNA libraries from mixed larval and adult tissues using gene-specific primers (provided in Additional file 1: Table S2) designed in MacVector 11.0.4 based on the sequences found in the transcriptome. PCR products were cloned into pGEM-T Easy vectors (Promega, USA) and then transformed into competent *Escherichia coli* cells. Plasmid DNA was isolated and sequenced in both forward and reverse directions using T7 and SP6 primers. Labeled antisense RNA probes were transcribed from linearized DNA using digoxigenin-11-UTP (Roche, USA) according to the manufacturer’s instructions.

### Head-specific genes

Additionally, we searched the transcriptome of *Ph. harmeri* in order to identify head-specific genes—*foxG*, *foxQ2* and *pax6*. We identified two potential paralogues of both *foxG* and *foxQ2* and called them correspondingly *foxGa*, *foxGb*, *foxQ2a* and *foxQ2b*. The vertebrate genes *pax4* and *pax6* originated through the vertebrate-specific duplication [123–125] and accordingly their protostome orthologue should be called *pax4/6*, same as in case of the other invertebrate Pax genes (*pax3/7*, *pax1/9* and *pax2/5/8*). Therefore, despite the fact that *pax6* is often used to refer to this gene in other protostomes, we decided to name the identified gene *pax4/6* in order to stress its co-orthology to both vertebrate genes [124]. We ran phylogenetic analyses to make sure that identified phoronid genes truly represent orthologues of the genes of interest. For Fox genes we aligned phoronid Fox sequences with a published alignment of Fox domains [97], while for Pax4/6 we assembled alignment from sequences available in GenBank (see Additional file 1 for alignments and list of used sequences). The alignments were trimmed in trimAl software [126] (using the gappyout option) and the phylogenetic trees were calculated with FastTree v2.1 [127] (using the LG amino acid substitution model). The obtained trees where visualized and adjusted in FigTree v1.4.3 and confirmed identity of all identified phoronid genes (Additional file 1: Figs. S3 and S4). Sequences of phoronid Fox genes and *pax4/6* were deposited in GenBank (accession numbers MN734372–MN734376). Probes against head-specific genes were synthesized in the same way as described for Hox genes (although we did not manage to clone *foxQ2a*). Additionally we used the same dig-labeled probes against *otx* and *six3/6* as in Andrikou et al. [85] (see “Method” section in there for more details regarding orthology of those genes).

### In situ hybridization and light microscopy

Single whole-mount in situ hybridization was performed following an established protocol [128] with proteinase K digestion time of 2 min. Probes were hybridized at a concentration of 1 ng/μl at 67 °C for approximately 72 h, detected with anti-digoxigenin-AP antibody in 1:5000 concentration in blocking buffer and visualized with nitroblue tetrazolium chloride and 5-bromo-4-chloro-3-indolyl phosphate. Embryos and larvae were mounted in 70% glycerol and examined with Zeiss Axiocam HRc connected to a Zeiss Axioscope Ax10 using bright-field Nomarski optics.

### Image processing and figure preparation

Light micrographs were adjusted in Adobe Photoshop CS6 for contrast and assembled in Adobe Illustrator CS6. All figures and drawings were prepared in Adobe Illustrator CS6.

## Supplementary information

**Additional file 1.** Additional figures and tables.

## Data Availability

Sequences generated and analyzed in this study have been deposited in NCBI’s GenBank database under accession numbers MN443105–MN443114 and MN734372–MN734376. All remaining data generated or analyzed during this study are included in this published article or its additional materials.
