# Supplementary material for: Hox gene expression during development of the phoronid Phoronopsis harmeri
Source: EvoDevo. 2020 Feb 10;11:2. doi: 10.1186/s13227-020-0148-z (PMC7011278; doi:10.1186/s13227-020-0148-z)
Supplement: Supplementary file 1 — Additional file 1. Additional figures and tables. [file 13227_2020_148_MOESM1_ESM.docx]

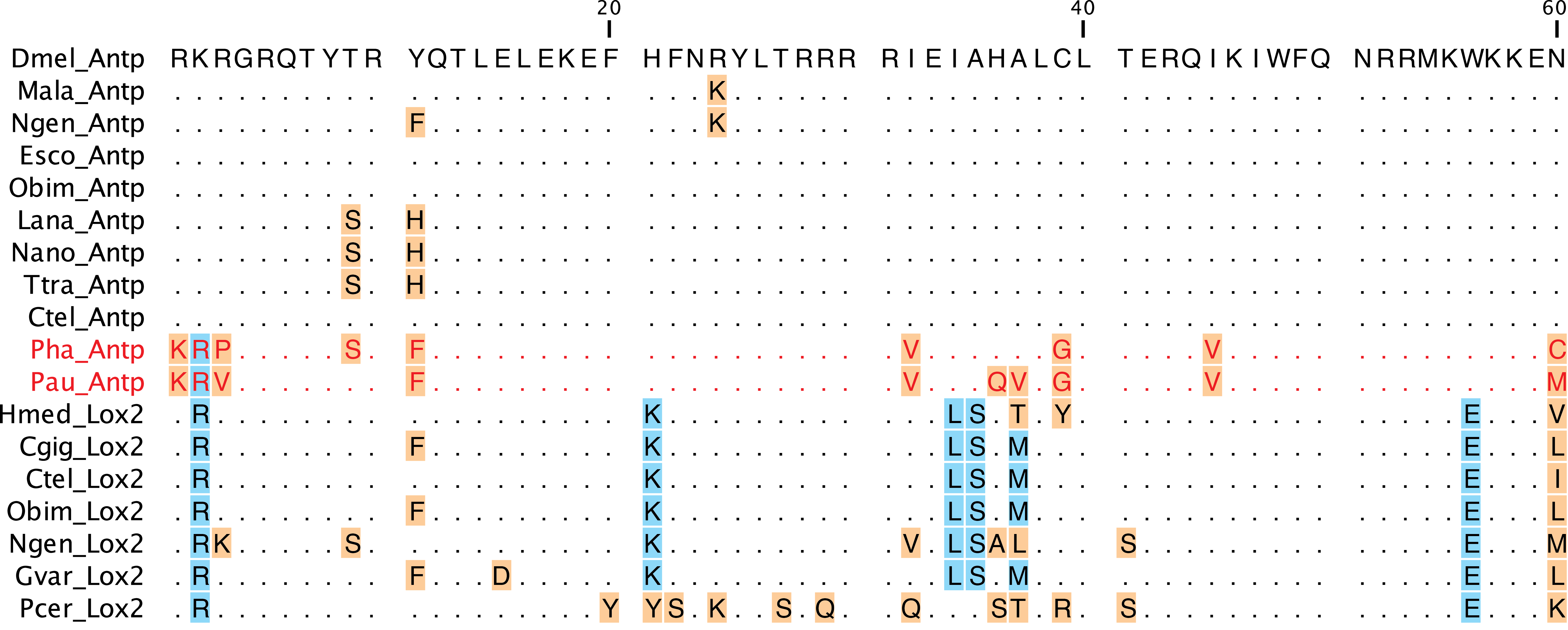


**Figure S1.** Sequences of homeodomains of spiralian *antp* and *lox2* aligned with the sequence of *Drosophila melanogaster* *antp*. Note that although sequences identified herein as phoronid *antp* share one amino acid with *lox2*, they lack remaining 5 signatures typical for *lox2*. Dots indicate identity to *D. melanogaster* *antp*, *lox2* specific signatures are highlighted in blue and remaining residues that differ from *D. melanogaster* *antp* are highlighted in orange. Phoronid sequences are in red. Dmel stands for *Drosophila melanogaster*, Hmed for *Hirudo medicinalis*, Pcer for *Piedicellina cernua*. Sequence of *PcerLox2* was obtained from GenBank (accession number KP691980). For remaining species abbreviations and source of sequences see Additional file 2: Tab. S1.

**
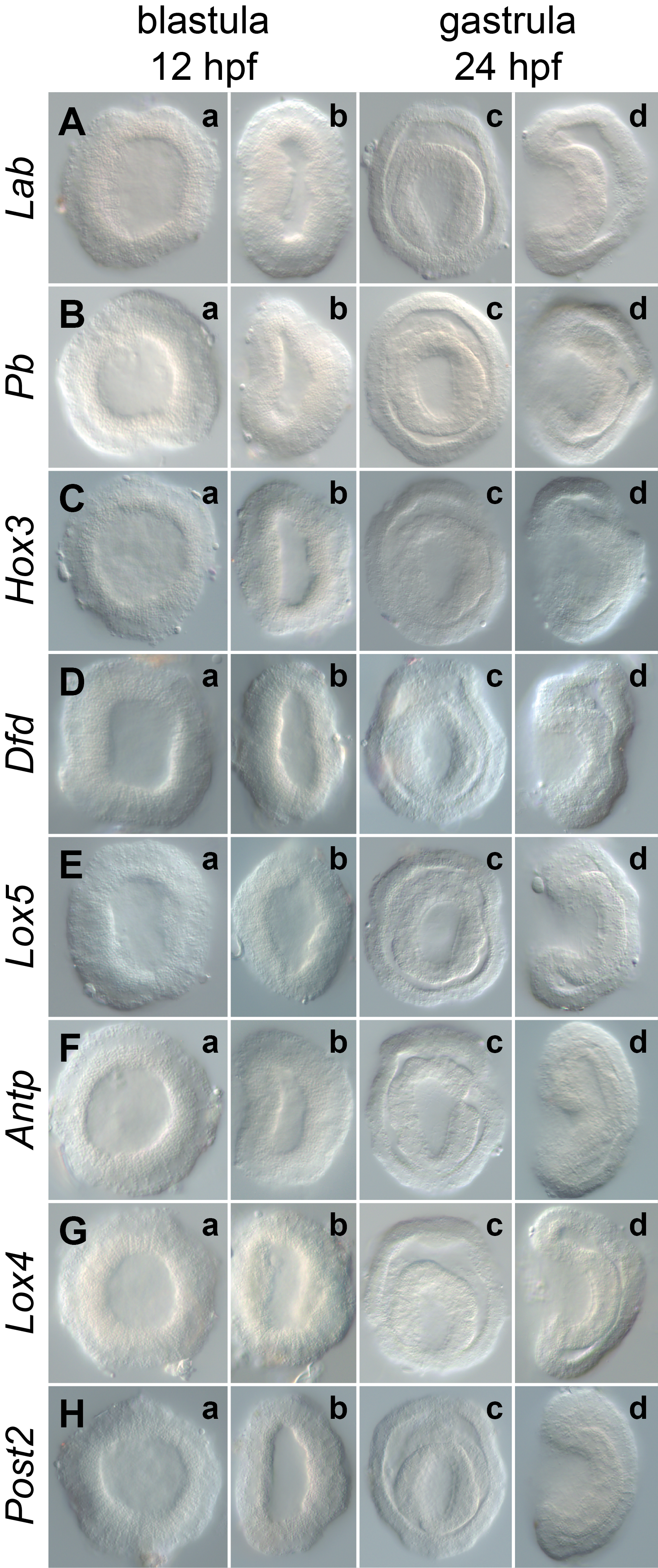
**

**Figure S2.** Lack of Hox gene expression in early developmental stages of *P. harmeri*. Name of each hybridized gene is shown on the left, while developmental stages are indicated on the top. Embryos on panels a and c are in the vegetal view, whereas embryos on panels b and d in the lateral view. Anterior is to the top on all panels. Photographs are not to scale.


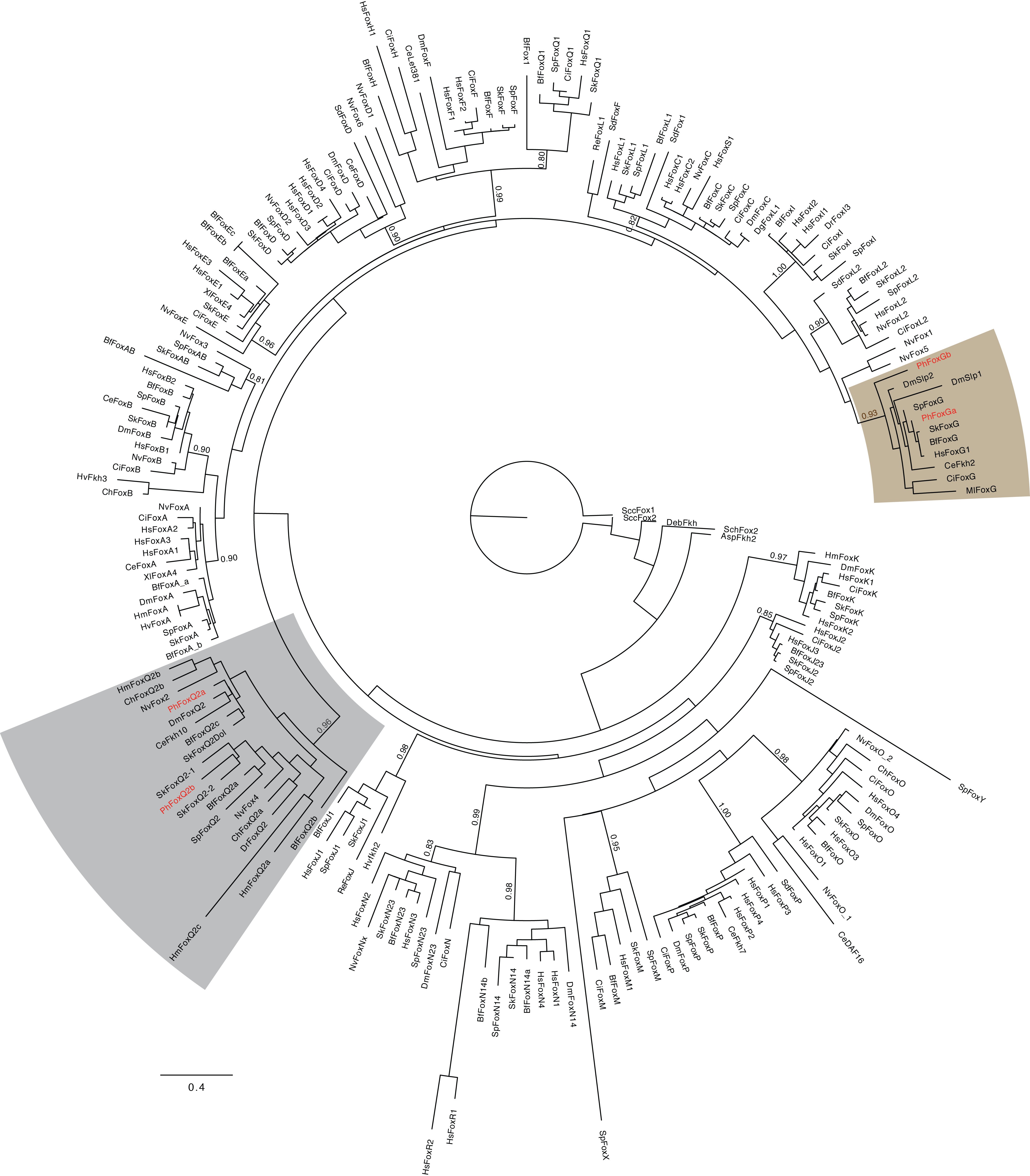


c

**Fig. S3**. Phylogenetic analysis of Fox sequences. SH-like support values are shown for the important nodes. FoxQ2 clade is coloured in grey and FoxG in brown. Scale bar on the lower left corner shows amino acid substitution rate per site. Genes from P. harmeri are marked in red. For abbreviation and source of other sequences see Fritzenwanker et al. 2014.


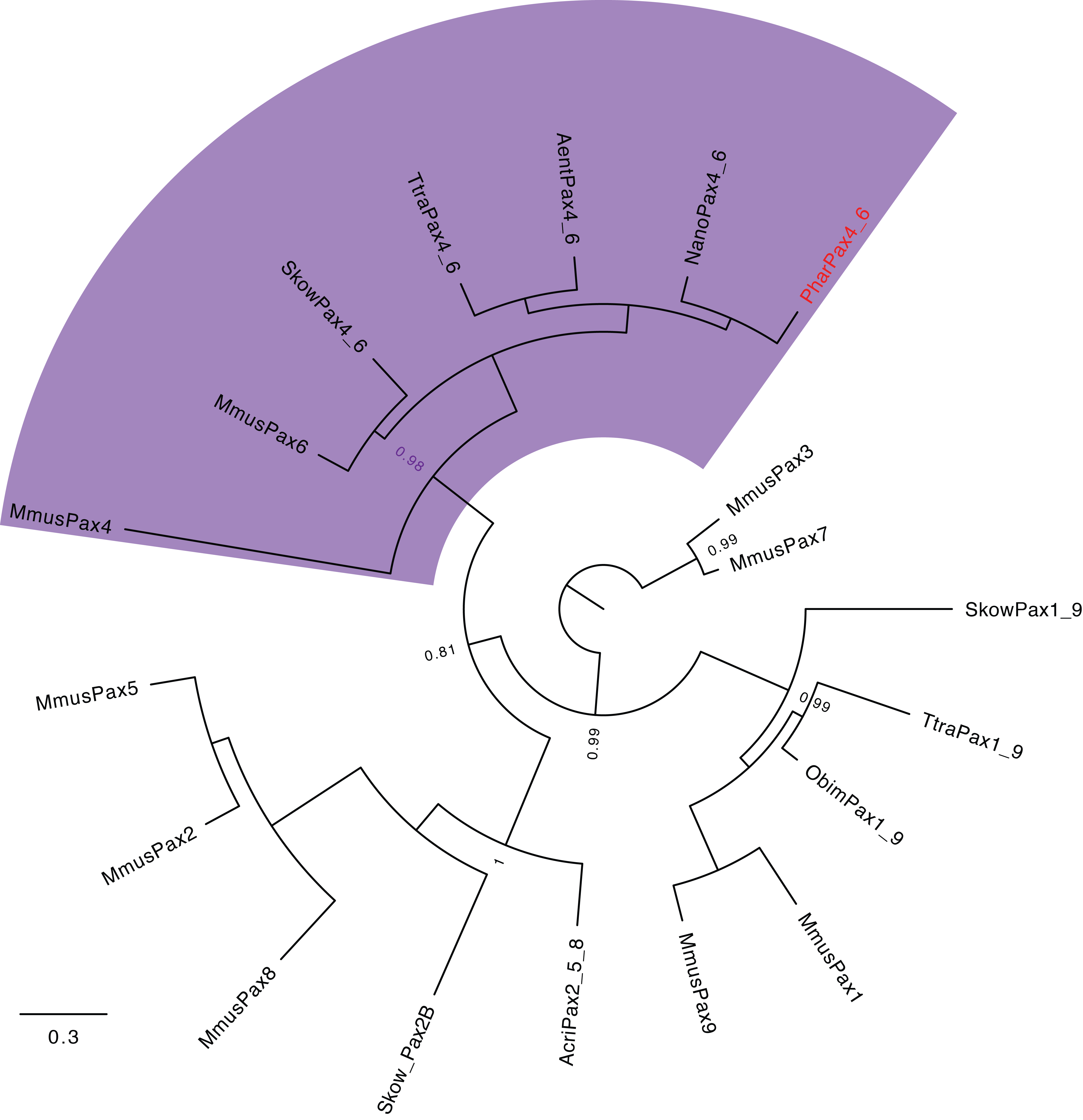


**Fig. S4**. Phylogenetic analysis of Pax sequences. SH-like support values are shown for the important nodes. Pax4/6 clade is colored in violet. Scale bar on the lower left corner shows amino acid substitution rate per site. Gene from *P. harmeri* is marked in red. For abbreviation and source of other sequences see table S3.

**Table S1.** Sequences used in the phylogenetic assessment of Hox genes orthology.

| **species** | **shortcut** | **clade** | **gene** | **accession no** | **source** |
| --- | --- | --- | --- | --- | --- |
| *Flaccisaggita enflata* | Fenf | Chaetognatha | Hox1 | ABS18809.1 | GenBank |
|  |  |  | Hox3 | ABS18810.1 |  |
|  |  |  | Hox4 | ABS18811.1 |  |
|  |  |  | Hox5 | ABS18812.1 |  |
|  |  |  | Hox6 | ABS18813.1 |  |
|  |  |  | Hox8 | ABS18814.1 |  |
|  |  |  | MedPost | ABS18817.1 |  |
|  |  |  | Posta | ABS18815.1 |  |
|  |  |  | Postb | ABS18816.1 |  |
| *Capitella teleta* | Ctel | Annelida | lab | ABY67952 | GenBank |
|  |  |  | pb | ABY67953 |  |
|  |  |  | Hox3 | ABY67954 |  |
|  |  |  | Dfd | ABY67955 |  |
|  |  |  | Scr | ABY67956 |  |
|  |  |  | Lox5 | ABY67957 |  |
|  |  |  | Antp | ABY67962 |  |
|  |  |  | Lox4 | ABY67958 |  |
|  |  |  | Lox2 | ABY67959 |  |
|  |  |  | Post1 | ABY67961 |  |
|  |  |  | Post2 | ABY67960 |  |
|  |  |  | Gsx | AAZ23124.1 |  |
|  |  |  | Cdx | AAZ95508 |  |
|  |  |  | Xlox | AAZ95509.1 |  |
|  |  |  | Evx1 | AFJ66236.1 |  |
|  |  |  | Evx2 | AFJ66237.1 |  |
| *Crassostrea gigas* | Cgig | Mollusca | Hox1 | CGI_10024083 | ENSEMBL |
|  |  |  | Hox2 | CGI_10024086 |  |
|  |  |  | Hox3 | CGI_10024087 |  |
|  |  |  | Hox4 | CGI_10024091 |  |
|  |  |  | Lox2 | CGI_10018592 |  |
|  |  |  | Lox4 | CGI_10026562 |  |
|  |  |  | Lox5 | CGI_10026565 |  |
|  |  |  | Evx | XP_011426639.1 | GenBank |
| *Octopus bimaculoides* | Obim | Mollusca | Hox1 | Ocbimv22030263 | ENSEMBL |
|  |  |  | Scr | Ocbimv22018468 |  |
|  |  |  | Antp | Ocbimv22036189 |  |
|  |  |  | Post1 | Ocbimv22015181 |  |
|  |  |  | Post2 | Ocbimv22031197 |  |
|  |  |  | Lox2 | Ocbimv22033340 |  |
|  |  |  | Lox4 | Ocbimv22009726 |  |
|  |  |  | Lox5 | Ocbimv22010205 |  |
| *Gibbula varia* | Gvar | Mollusca | HoxA | ACX84671.1 | GenBank |
|  |  |  | Hox2 | ADJ18233.1 |  |
|  |  |  | Hox3 | ADJ18232.1 |  |
|  |  |  | Hox4 | ACX84672.1 |  |
|  |  |  | Hox5 | ADJ18234.1 |  |
|  |  |  | Lox5 | ADJ18235.1 |  |
|  |  |  | Hox7 | ADJ18236.1 |  |
|  |  |  | Lox2 | ADJ18238.1 |  |
|  |  |  | Lox4 | ADJ18237.1 |  |
|  |  |  | Post1 | ACX84673.1 |  |
| *Euprymna scolopes* | Esco | Mollusca | lab | AY330184 | GenBank |
|  |  |  | Hox3 | AY330185 |  |
|  |  |  | Scr | AY330186 |  |
|  |  |  | Lox5 | AY330187 |  |
|  |  |  | Antp | AY330188 |  |
|  |  |  | Lox4 | AY330189 |  |
|  |  |  | Post1 | AAL25811.1 |  |
|  |  |  | Post2 | AY330191 |  |
| *Micrura alaskensis* | Mala | Nemertea | lab | KP762174 | GenBank |
|  |  |  | pb | KP762176 |  |
|  |  |  | Hox3 | KP762173 |  |
|  |  |  | Dfd | KP762180 |  |
|  |  |  | Scr | KP762177 |  |
|  |  |  | Lox5 | KP762179 |  |
|  |  |  | Antp | KP762171 |  |
|  |  |  | Lox4 | KP762175 |  |
|  |  |  | Post2 | KP762178 |  |
| *Notospermus geniculatus* | Ngen | Nemertea | labA | g12075.t1 | marinegenomics.oist.jp |
|  |  |  | labB | g24513.t1 |  |
|  |  |  | pbA | g6836.t1 |  |
|  |  |  | pbB | g12074.t1 |  |
|  |  |  | Hox3A | g30654.t1 |  |
|  |  |  | Hox3B | g6837.t1 |  |
|  |  |  | ScrA | g19273.t1 |  |
|  |  |  | ScrB | g35291.t1 |  |
|  |  |  | DfdA | g33684.t1 |  |
|  |  |  | DfdB | g35826.t1 |  |
|  |  |  | Antp | g19270.t1 |  |
|  |  |  | Lox5 | g19271.t1 |  |
|  |  |  | Lox2 | g26697.t1 |  |
|  |  |  | Lox4 | g26698.t1 |  |
|  |  |  | Post2A | g26696.t1 |  |
|  |  |  | Post2B | g16861.t1 |  |
| *Crisularia turrita* | Ctur | Bryozoa | pb | AAS77225 | GenBank |
|  |  |  | Hox3 | AAS77226 |  |
|  |  |  | Dfd-a | AAS77227 |  |
|  |  |  | Dfd-b | AAS77228 |  |
|  |  |  | Lox5 | AAS77229 |  |
|  |  |  | Post2 | AAS77230 |  |
| *Lingula anatina* | Lana | Brachiopoda | lab | g10891 | ENSEMBL |
|  |  |  | pb | g10890 |  |
|  |  |  | Hox3 | g10889 |  |
|  |  |  | Dfd | g10888 |  |
|  |  |  | Scr | g10887 |  |
|  |  |  | Lox5 | g10886 |  |
|  |  |  | Antp | g10892 |  |
|  |  |  | Post1 | g12396 |  |
|  |  |  | Post2 | g12399 |  |
|  |  |  | Evx | XP_013401616.1 | GenBank |
| *Novocrania anomala* | Nana | Brachiopoda | lab | KX372756 | GenBank |
|  |  |  | pb | KX372757 |  |
|  |  |  | Hox3 | KX372758 |  |
|  |  |  | Scr | KX372759 |  |
|  |  |  | Lox5 | KX372760 |  |
|  |  |  | Dfd | KX372769 |  |
|  |  |  | Antp | KX372770 |  |
|  |  |  | Lox4 | KX372773 |  |
|  |  |  | Post2 | KX372774 |  |
|  |  |  | Evx | AHY88454.1 |  |
| *Terebratalia transversa* | Ttra | Brachiopoda | lab | KX372761 | GenBank |
|  |  |  | pb | KX372762 |  |
|  |  |  | Hox3 | KX372763 |  |
|  |  |  | Dfd | KX372764 |  |
|  |  |  | Scr | KX372765 |  |
|  |  |  | Lox5 | KX372766 |  |
|  |  |  | Lox4 | KX372767 |  |
|  |  |  | Post2 | KX372768 |  |
|  |  |  | Antp | KX372771 |  |
|  |  |  | Post1 | KX372772 |  |
|  |  |  | Evx | AHY88463.1 |  |
| *Phoronis australis* | Pau | Phoronida | lab | g5412.t1 | marinegenomics.oist.jp |
|  |  |  | pb | g5413.t1 |  |
|  |  |  | Hox3 | g5414.t1 |  |
|  |  |  | Dfd | g5415.t1 |  |
|  |  |  | Lox5 | g5416.t1 |  |
|  |  |  | Lox4 | g5418.t1 |  |
|  |  |  | Antp | g5417.t1 |  |
|  |  |  | Post2 | g5419.t1 |  |
| *Phoronopsis harmeri* | Pha | Phoronida | lab |  | this study |
|  |  |  | pb |  |  |
|  |  |  | Hox3 |  |  |
|  |  |  | Dfd |  |  |
|  |  |  | Lox5 |  |  |
|  |  |  | Lox4 |  |  |
|  |  |  | Antp |  |  |
|  |  |  | Post2 |  |  |

**Table S2.** Primers used to clone genes from cDNA libraries

| **gene** | **forward** |  | **reverse** |
| --- | --- | --- | --- |
| lab | AGTCAAAACCTGAATCTCTCACACG | GCCCGAACAAGAACACCGAAG |  |
| pb | CATCCAAGCCACACAGTGAAGC | GGTTTGTCCCGAATAGTCTGGTG |  |
| hox3 | GACAACCGCCTCATCCGATAG | CGACACAGTGACTCCTGACACG |  |
| dfd | TGTCGCCTCCCCATCACTCACCAAG | TCAGAGCATCTATTTGAAAACCTGC |  |
| lox5 | GGACTCGCAGCAAACTTATCTTCAC | TTCCATTTCATCCGCCTGTTC |  |
| antp | GCAGAAAAGAAAAGACCAGGACG | AACCCGCACAGTATTATGACCTTG |  |
| lox4 | CGTCTTGCCCTCGTTAGTTCAC | CTCGTTCTCCACATAAGCAAGGAC |  |
| post2 | TGGGCACCTGTGGCTTACTG | TTTTCTTCCTTTTCATCCGTCG |  |

**TableS3.** Sequences used in the phylogenetic assessment of Pax genes orthology

| **species** | **shortcut** | **gene** | **accession no** |
| --- | --- | --- | --- |
| *Mus musculus* | Mmus | pax4 | BAA24517.1 |
| *Mus musculus* | Mmus | pax6 | AAH36957.1 |
| *Antalis entalis* | Aent | pax4/6 | QFU47189.1 |
| *Saccoglossus kowalevskii* | Skow | pax4/6 | AAP79294.1 |
| *Novocrania anomala* | Nano | pax4/6 | ALS19770.1 |
| *Terebratalia transversa* | Ttra | pax4/6 | ALS19761.1 |
| *Mus musculus* | Mmus | pax3 | AAH48699.1 |
| *Mus musculus* | Mmus | pax7 | AAG16663.3 |
| *Mus musculus* | Mmus | pax1 | XP_006498974.1 |
| *Terebratalia transversa* | Ttra | pax1/9 | AJV21320.1 |
| *Octopus bimaculoides* | Obim | pax1/9 | ACR19860.1 |
| *Saccoglossus kowalevskii* | Skow | pax1/9 | NP_001158408.1 |
| *Mus musculus* | Mmus | pax9 | NP_035171.1 |
| *Mus musculus* | Mmus | pax2 | CAA39302.1 |
| *Saccoglossus kowalevskii* | Skow | pax2B | XP_006813069.1 |
| *Acanthochitona crinita* | Acri | pax2/5/8 | ALM30867.1 |
| *Mus musculus* | Mmus | pax5 | NP_032808.1 |
| *Mus musculus* | Mmus | pax8 | CAA40725.1 |

**Alignment of Hox sequences used for phylogenetic analysis (Fig. 2) in FASTA format:**

>Lana_Lox5

EIGYEQKRTRQTYTRYQTLELEKEFHYNRYLTRRRRIEIAHHLGLTERQIKIWFQNRRMKWKKENNIPKLTGP

>Nano_Lox5

DIGYEQKRTRQTYTRYQTLELEKEFHYNRYLTRRRRIEIAHALGLTERQIKIWFQNRRMKWKKENNIAKLTGP

>Pau_Lox5

EIGYEQKRTRQTYTRFQTLELEKEFHYNRYLTRRRRIEIAHALGLTERQIKIWFQNRRMKWKKENNVPKLTGP

>Pha_Lox5

EIGYEQKRTRQTYTRFQTLELEKEFHYNRYLTRRRRIEIAHSLGLTERQIKIWFQNRRMKWKKENNVPKLTGP

>Ttra_Lox5

DIGYEQKRTRQTYTRYQTLELEKEFHFNRYLTRRRRIEIAHALGLTERQIKIWFQNRRMKWKKENNLPKLTGP

>Ctel_Lox5 [Capitella teleta]

DFGYEQKRTRQTYTRYQTLELEKEFHYNRYLTRRRRIEIAHALQLTERQIKIWFQNRRMKYKKENNISKLTGP

>Mala_Lox5

EMPIEQKRTRQTYTRYQTLELEKEFHFNKYLTRRRRIEIAHALGLTERQIKIWFQNRRMKWKKENNLQKLTGP

>Ngen_Lox5

EMPIEQKRTRQTYTRYQTLELEKEFHFNKYLTRRRRIEIAHALGLSERQIKIWFQNRRMKWKKENNLQKLTGP

>Esco_Antp

QYGPHRKRGRQTYTRYQTLELEKEFHFNRYLTRRRRIEIAHALCLTERQIKIWFQNRRMKWKKENKAEMPGTE

>Mala_Antp

QFGPDRKRGRQTYTRYQTLELEKEFHFNKYLTRRRRIEIAHALCLTERQIKIWFQNRRMKWKKENKPSEGGTS

>Ngen_Antp

QFGPDRKRGRQTYTRFQTLELEKEFHFNKYLTRRRRIEIAHALCLTERQIKIWFQNRRMKWKKENKQPNGPNC

>Cgig_Lox5

EVTYEQKRTRQTYTRYQTLELEKEFHFNRYLTRRRRIEIAHLLGLTERQIKIWFQNRRMKWKKDNNIPKLTGP

>Esco_Lox5

ETAYEQKRTRQTYTRFQTLELEKEFHFNRYLTRRRRIEIAHSLGLSERQIKIWFQNRRMKWKKENNVSKLTGP

>Gvar_Hox7 [Gibbula varia]

DVHFEQKRTRQTYTRYQTLELEKEFHFNRYLTRRRRIEVAHMLGLTERQIKIWFQNRRMKWKKENNVSKLTGP

>Gvar_Lox5 [Gibbula varia]

DVHFEQKRTRQTYTRYQTLELEKEFHFNRYLTRRRRIEVAHMLGLTERQIKIWFQNRRMKWKKDNNVSKVTGP

>Ngen_ScrA

---IE-KRTRTSYTRYQTLELEKEFHFNRYLTRRRRIEIAHALNLTERQIKIWFQNRRMKWKKEQKLAHITKS

>Ngen_ScrB

---IE-KRTRTSYTRYQTLELEKEFHFNRYLTRRRRIEIAHALNLTERQIKIWFQNRRMKWKKEQKLAHITKS

>Mala_Scr

---MESKRTRTSYTRYQTLELEKEFHFNRYLTRRRRIEIAHALNLTERQIKIWFQNRRMKWKKEQKLAHITKS

>Lana_Scr

---IESKRTRTSYTRHQTLELEKEFHFNRYLTRRRRIEIAHALNLTERQIKIWFQNRRMKWKKEQKLAHLTKT

>Nano_Scr

----DNKRTRTSYTRHQTLELEKEFHFNRYLTRRRRIEIAHALNLTERQIKIWFQNRRMKWKKEQKLAHLTKT

>Ttra_Scr

--NAESKRTRTSYTRHQTLELEKEFHFNRYLTRRRRIEIAHALNLTERQIKIWFQNRRMKWKKEQKVSHITKN

>Ctel_Scr [Capitella teleta]

---ADNKRTRTSYTRHQTLELEKEFHFNRYLTRRRRIEIAHSLNLTERQIKIWFQNRRMKWKKEHKLAHLAKS

>Ctur_Lox5 [Crisularia turrita]

--GYEQKRTRQTYTRYQTLELEKEFHYNRYLTRRRRIEIAHTLGLTERQIKIWFQNRRMKWKKENNIAKLTG-

>Fenf_Hox6 [Flaccisagitta enflata]

---FDRKRGRQTYTRYQTLELEKEFHFNRYLTRRRRIDIAHALCLTERQIKIWFQNRRMKWKKEQKAALGVGM

>Obim_Antp

---PHRKRGRQTYTRYQTLELEKEFHFNRYLTRRRRIEIAHALCLTERQIKIWFQNRRMKWKKENKAEVPVSE

>Fenf_Hox5 [Flaccisagitta enflata]

DLGIDQKRTRQTYTRHQTLELEKEFHFNRYLTRRRRIEIVHALGLTERQIKIWFQNRRMKWKKENNLKSINDA

>Nano_Dfd

YNGLEPKRSRTAYTRHQILELEKEFHFNRYLTRRRRIEIAHALCLTERQIKIWFQNRRMKWKKEHKLPNTKTR

>Ttra_Dfd

YNGLEPKRSRTAYTRHQILELEKEFHFNRYLTRRRRIEIAHALCLTERQIKIWFQNRRMKWKKEHKLPNTKTR

>Pau_Dfd

YNGLESKRTRTAYTRHQILELEKEFHFNRYLTRRRRIEIAHALCLTERQIKIWFQNRRMKWKKEHKLPNTKTR

>Pha_Dfd

YNGMESKRTRTAYTRHQILELEKEFHFNRYLTRRRRIEIAHALCLTERQIKIWFQNRRMKWKKEHKLPNTKTR

>Lana_Dfd

YNGLEPKRSRTAYTRHQILELEKEFHFNRYLTRRRRIEIAHSLCLTERQIKIWFQNRRMKWKKEHKLPNTKNK

>Mala_Dfd

FNGGENKRTRTAYTRHQILELEKEFHFNRYLTRRRRIEIAHALCLTERQIKIWFQNRRMKWKKEHKLPNTKLR

>Ngen_DfdA

FSSGENKRTRTAYTRHQILELEKEFHFNRYLTRRRRIEIAHALCLTERQIKIWFQNRRMKWKKEHKLPNTKLR

>Ngen_DfdB

FSSGENKRTRTAYTRHQILELEKEFHFNRYLTRRRRIEIAHALCLTERQIKIWFQNRRMKWKKEHKLPNTKLR

>Cgig_Hox4

SLVSESKRNRTAYTRHQILELEKEFHFNRYLTRRRRIEIAHTLCLSERQIKIWFQNRRMKWKKEHKLPNTKTR

>Ctel_Dfd [Capitella teleta]

----DSKRTRTAYTRHQILELEKEFHFNRYLTRRRRIEIAHTLCLSERQIKIWFQNRRMKWKKEHKLPNTKTR

>Fenf_Hox4 [Flaccisagitta enflata]

NFAGEPKRARTAYTRHQVLELEKEFHFNRYLTRRRRIEIAHALCLTERQIKIWFQNRRMKWKKDHKLPNTKTV

>Lana_Antp

---PDRKRGRQTYSRHQTLELEKEFHFNRYLTRRRRIEIAHALCLTERQIKIWFQNRRMKWKKENKGIELSRD

>Nano_Antp

QFGPDRKRGRQTYSRHQTLELEKEFHFNRYLTRRRRIEIAHALCLTERQIKIWFQNRRMKWKKENRGADLQRD

>Ttra_Antp

G-GPDRKRGRQTYSRHQTLELEKEFHFNRYLTRRRRIEIAHALCLTERQIKIWFQNRRMKWKKENKQEESMKN

>Ctur_Dfda [Crisularia turrita]

--GLDPKRARTAYTRHQILELEKEFHFNRYLTRRRRIEIAHTLDLSERQIKIWFQNRRMKWKKEHKLPNTKGK

>Esco_Scr

P-DGESKRSRTSYTRHQTLELEKEFHYNKYLTRRRRIEIAHALNLTERQIKIWFQNRRMKWKKEHKLSHIAKN

>Obim_Scr

P-DGESKRSRTSYTRHQTLELEKEFHYNKYLTRRRRIEIAHALNLTERQIKIWFQNRRMKWKKEHKLSHIAKN

>Gvar_Hox5 [Gibbula varia]

GNDADSKRSRTSYTRHQTLELEKEFHYNKYLTRRRRIEIAHALNLTERQIKIWFQNRRMKWKKDHKLSHIAKN

>Ctel_Antp [Capitella teleta]

KAGPERKRGRQTYTRYQTLELEKEFHFNRYLTRRRRIEIAHALCLTERQIKIWFQNRRMKWKKENRQIEVLRQ

>Fenf_Hox8 [Flaccisagitta enflata]

----PRKRGRQTYTRYQTLELEKEFHFNRYLTRRRRIEMAHALCLTERQIKIWFQNRRMKEKKEKQKIEEMKV

>Mala_Lox4

PNSAQRRRGRQTYSRYQTLELEKEFQFNHYLTRRRRIEIAHSLCLTERQIKIWFQNRRMKLKKERQQIKELND

>Ngen_Lox4

PNSAQRRRGRQTYSRYQTLELEKEFQFNHYLTRRRRIEIAHSLCLTERQIKIWFQNRRMKLKKERQQIKELND

>Ttra_Lox4

PNSAQRRRGRQTYSRYQTLELEKEFQFNHYLTRKRRIEVAHALCLTERQIKIWFQNRRMKLKKERQQIKELND

>Cgig_Lox4

PNSAHRRRGRQTYSRYQTLELEKEFQFNHYLTRKRRIEVAHSLCLTERQIKIWFQNRRMKLKKERQAIKEIND

>Ctel_Lox4 [Capitella teleta]

PNSSQRRRGRQTYSRYQTLELEKEFQFNHYLTRKRRIEIAHALCLTERQIKIWFQNRRMKLKKERQQIKDLN-

>Pha_Lox4

PNSAQRRRGRQTYTRYQTLELEKEFQFNHYLTRKRRIEIAHVLCLTERQIKIWFQNRRMKLKKERQQIKEMNE

>Pau_Lox4

PNSAQRRRGRQTYSRYQTLELEKEFQFNNYLTRKRRIEIAHTLRLTERQVKIWFQNRRMKLKKEKQQIKEIN-

>Nano_Lox4

PNSAQRRRGRQTYSRFQTLELEKEFQFNHYLTRKRRIEVAHALCLTERQIKIWFQNRRMKLKKERQQIKEMNE

>Cgig_Lox2

PNSNQRRRGRQTYTRFQTLELEKEFKFNRYLTRRRRIELSHMLCLTERQIKIWFQNRRMKEKKELQAIKELNE

>Ctel_Lox2 [Capitella teleta]

PNSNQRRRGRQTYTRYQTLELEKEFKFNRYLTRRRRIELSHMLCLTERQIKIWFQNRRMKEKKEIQAIKELNE

>Obim_Lox2

PNSNQRRRGRQTYTRFQTLELEKEFKFNRYLTRRRRIELSHMLCLTERQIKIWFQNRRMKEKKELQAIKELNE

>Ngen_Lox2

PNSNQRRKGRQTYSRYQTLELEKEFKFNRYLTRRRRVELSALLCLSERQIKIWFQNRRMKEKKEMQAIKELNK

>Gvar_Lox2 [Gibbula varia]

QKSNQRRRGRQTYTRFQTLDLEKEFKFNRYLTRRRRIELSHMLCLTERQIKIWFQNRRMKEKKELQAIKELNS

>Gvar_Lox4 [Gibbula varia]

PNSAQRRRGRQTYSRYQTLELEKEFQFNHYLTRKRRIEIAHTLCLTERQIKIWFQNRRMKMKKERQAIKDING

>Esco_Lox4

PNSSQRRRGRQTYSRFQTLELEKEFQYNNYLTRKRRIEVAHALNLSERQIKIWFQNRRMKLKKEKQQIREMNG

>Obim_Lox4

PNSSQRRRGRQTYSRFQTLELEKEFQYNNYLTRKRRIEVAHALNLSERQIKIWFQNRRMKLKKEKQQIRELNV

>Gvar_Hox4 [Gibbula varia]

--NGEYKRTRTAYTRHQVLELGKEFHFNRYLIRRRRIEITHTLCLSERQIKIWFQNRRMKWKKEHKLPNTKTG

>Pha_Antp

QSAEKKRPGRQTYSRFQTLELEKEFHFNRYLTRRRRVEIAHALGLTERQVKIWFQNRRMKWKKECKALKNMND

>Pau_Antp

--TEKKRVGRQTYTRFQTLELEKEFHFNRYLTRRRRVEIAQVLGLTERQVKIWFQNRRMKWKKEMKALKSLNE

>Ctur_Dfdb [Crisularia turrita]

--DGDNKRTRTAYTRQQVLELEKEFHYNRYLTQRRRIEIAHTLTLSERQIKIWFQNRRMKWKKDHKLSSSKGR

>Obim_Lox5

----------------------------------RRIEIAHSLGLSERQIKIWFQNRRMKWKKENNVQKLTGP

>Nano_Hox3

-----SKRARTAYTSAQLVELEKEFHFNRYLCRPRRIEMAALLSLTERQIKIWFQNRRMKFKKEQKQKVLMEK

>Ttra_Hox3

-----SKRARTAYTSAQLVELEKEFHFNRYLCRPRRIEMAALLSLSERQIKIWFQNRRMKFKKEQKQKAILEK

>Lana_Hox3

-----TKRARTAYTSAQLVELEKEFHFNRYLCRPRRIEMAALLNLTERQIKIWFQNRRMKFKKEQKQKVMLEK

>Pau_Hox3

-----AKRARTAYTSAQLVELEKEFHFNRYLCRPRRIEMAALLNLSERQIKIWFQNRRMKFKKEQKQKIAVAK

>Pha_Hox3

-----AKRARTAYTSAQLVELEKEFHFNRYLCRPRRIEMAALLNLSERQIKIWFQNRRMKFKKEQKQKVALEK

>Mala_Hox3

-----PKRSRTAYTSAQLVELEKEFHFNRYLCRPRRIEMAALLNLSERQIKIWFQNRRMKYKKDQKQKNLMEK

>Ngen_Hox3A

-----PKRSRTAYTSAQLVELEKEFHFNRYLCRPRRIEMAALLNLSERQIKIWFQNRRMKYKKDQKQKNLMEK

>Ngen_Hox3B

-----PKRSRTAYTSAQLVELEKEFHFNRYLCRPRRIEMAALLNLSERQIKIWFQNRRMKYKKDQKQKNLMEK

>Ctel_Hox3 [Capitella teleta]

-----SKRARTAYTSAQLVELEKEFHFNRYLCRPRRIEMAALLNLTERQIKIWFQNRRMKYKKDQKQKNLMEK

>Cgig_Hox3

--PEPTKRARTAYTSAQLVELEKEFHFNRYLCRPRRIEMAALLSLTERQIKIWFQNRRMKFKKEQRQKPHSEK

>Esco_Hox3

--EQPAKRARTAYTSAQLVELEKEFHFNQYLCRPRRIEMAALLNLSERQIKIWFQNRRMRFKKEKKLKVNMDK

>Gvar_Hox3 [Gibbula varia]

--VEPATRARTAYTSAQLVELEKEFHFNRYLCRPRRIEMAALLNLSERQIKIWFQNRRMKFKKDCRLKGGSDK

>Ngen_PbA

--GNNPRRLRTAYTNSQLLELEKEFHFNKYLCRPRRIEIAAALDLTERQVKVWFQNRRMKFKRQTGKGDKGEG

>Ngen_PbB

--GNNPRRLRTAYTNSQLLELEKEFHFNKYLCRPRRIEIAAALDLTERQVKVWFQNRRMKFKRQTGKGDKGDG

>Mala_Pb

--GNNPRRLRTAYTNSQLLELEKEFHFNKYLCRPRRIEIAASLDLTERQVKVWFQNRRMKYKRQSGK----DG

>Lana_Pb

--KQHTRRLRTAYTNTQLLELEKEFHFNKYLCRPRRIEIAASLDLTERQVKVWFQNRRMKFKRQSQLKQQDAP

>Nano_Pb

--SNHARRLRTAYTNTQLLELEKEFHFNKYLCRPRRIEIAASLDLTERQIKVWFQNRRMKFKRQSQLKHGDGS

>Gvar_Hox2 [Gibbula varia]

--AGCSRRLRTAYTNTQLLELEKEFHFNKYLCRPRRIEIAASLDLTERQIKVWFQNRRMKYKRQSQS---GRS

>Ttra_Pb

--PNHPRRLRTAYTNTQLLELEKEFHFNKYLCRPRRIEIAASLDLTERQIKVWFQNRRMKFKRQSQNKSDGSG

>Cgig_Hox2

--GGGTRRLRTAYTNTQLLELEKEFHFNKYLCRPRRIEIAASLDLTERQIKVWFQNRRMKYKRQTQSQRQKAE

>Pha_Pb

--CTSQRRLRTAYTNTQLLELEKEFHFNKYLCRPRRIEIAASLDLTERQIKVWFQNRRMKFKRQKQG------

>Ctel_Pb [Capitella teleta]

--SNHPRRLRTAYTNTQLLELEKEFHFNKYLCRPRRIEIAASLDLTERQIKVWFQNRRMKFKRQTGKGSGNSP

>Pau_Pb

--AISQRRLRTAYTNTQLLELEKEFHFNKYLCRPRRIEIAASLDLSERQIKVWFQNRRMKFKRQKQAGHGNRK

>Ctel_Xlox [Capitella teleta]

QTFSENKRTRTAYTRAQLLELEKEFHFNRYITRPRRVELAAHLNLTEQHIKIWFQNRRMKWKKDVDKKRPQQS

>Fenf_MedPost [Flaccisagitta enflata]

---TPHRERRQTYTRHQTAELEREYVTNRYLTRRRRIEISQSLHLSERQIKIWFQNRRMKEKREKDHVTSPRK

>Mala_Lab

--AGQPNTGRTNFTNKQLTELEKEFHFNKYLTRARRIEIAAALGLNERQIKIWFQNRRMKQKKRMKEGLVQNN

>Ngen_LabA

--AGQPNTGRTNFTNKQLTELEKEFHFNKYLTRARRIEIAAALGLNERQIKIWFQNRRMKQKKRMKEGLVQNN

>Ngen_LabB

--AGQPNTGRTNFTNKQLTELEKEFHFNKYLTRARRIEIAAALGLNERQIKIWFQNRRMKQKKRMKEGLVQNN

>Lana_Lab

--GNIPNMGRTNFSNKQLTELEKEFHFNKYLTRARRIEIAAALGLNERQIKIWFQNRRMKQKKRMKESQTLP-

>Nano_lab

--ANAPNMGRTNFSNKQLTELEKEFHFNKYLTRARRIEIAAALGLNERQIKIWFQNRRMKQKKRLKEGGSMT-

>Ttra_Lab

--NQAPNMGRTNFSNKQLTELEKEFHFNKYLTRARRIEIAAALGLNERQIKIWFQNRRMKQKKRMKESPLSAH

>Pau_Lab

--SAASNMGRTNFTNKQLTELEKEFHFNKYLTRARRIEIAATLGLNERQIKIWFQNRRMKQKKRMKECRAQMK

>Pha_Lab

--TTATNMGRTNFTNKQLTELEKEFHFNKYLTRARRIEIAATLGLNERQIKIWFQNRRMKQKKRMKECRAQMK

>Ctel_Lab [Capitella teleta]

--AGQPNMGRTNFTNKQLTELEKEFHFNKYLTRARRIEIAASLGLNERQIKIWFQNRRMKQKKRLKENTSTTP

>Fenf_Hox1 [Flaccisagitta enflata]

--GGINNTGRTNFTTKQLTELEKEFHFNKYLTRARRIEIAGALQLNERQIKIWFQNRRMKQKKRMKEGLIPPD

>Gvar_Hox1 [Gibbula varia]

--GGINSTGRTNFWNKQAFEFEKEFHFNKYFTRARRIEIAAALGLNERQIKIWFQNSRMKQKKRMSEIQFEKG

>Cgig_Hox1

--TPQPNMGRTNFTNKQLTELEKEFHFNKYLTRARRIEIAAALGLNERQIKIWFQNRRMKQKKRLREAQFDQN

>Obim_Hox1

--VGGGGTGRTNFTNKQLTELEKEFHFNKYLTRARRIEIAAALGLNETQVKIWFQNRRMKQKKRLKEAQGTTG

>Esco_Lab

--GGGNSTGRTNFTNKQLTELEKEFHFNKYLTRARRIEIAAA-------------------------------

>Pha_Xlox

--------------------LEKEFHFNKYISRPRRIELAALLNLTERHIKIWFQNRRMKWKKDEAKRRPRPL

>Ctel_Gsx [Capitella teleta]

ESSDAVKRMRTAFSSTQLLELEREFASNMYLSRLRRIEIATYLSLSEKQVKIWFQNRRVKFKKEGAAHGSRDH

>Pha_Gsx

DDLDSSKRIRTAFTSTQLLELEREFAANMYLSRLRRIEIATYLNLSEKQVKIWFQNRRVKYKKEGG--GSRDR

>Esco_Post1

PSAIALRKRRRPYSKYQIAELEREYALSTYISKSRRWELSQLLNLSERQIKIWFQNRRIKAKKLQKRDETLKT

>Obim_Post1

PSAIALRKRRRPYSKYQIAELEREYAISTYISKSRRWELSQLLNLSERQIKIWFQNRRIKAKKLQKRDETLKG

>Gvar_Post1 [Gibbula varia]

PTTVTLRKRRRPYSKFQIAELEREYN-GSYVSESRRWELSQLINLSERQIKIWFQNRRIKAKKIIKRDDISPQ

>Lana_Post1

PAVIHMRKKRKPYSKYQIAELEREYVSNTYISKPKRWELSQRLQLSERQIKIWFQNRRMKEKKVKGGKQT---

>Ttra_Post1

PTVIHMRKRRKPYSKQQINELEREYVKTTYISKPKRWELAQRLNLSERQIKIWFQNRRMKEKKMRGSRRX---

>Ctur_Pb [Crisularia turrita]

------------------------FHFNKYLCRPRRIEIAASLDLTERQIKV---------------------

>Ctur_Hox3 [Crisularia turrita]

------------------------FHFNRYLCRPRRIEMAALLSLTERQIKI---------------------

>Ngen_Post2A

---PRTRKKRKPYTRYQTMVLENEFMTNSYITRQKRWEISCKLHLSERQIKVWFQNRRMKRKKLNARTK-IKS

>Ngen_Post2B

---PRTRKKRKPYTRYQTMVLENEFMTNSYITRQKRWEISCKLHLSERQIKVWFQNRRMKRKKLNARTK-IKS

>Mala_Post2

---PRTRKKRKPYTRYQTMVLENEFMTNSYITRQKRWEISCKLHLTERQIKVWFQNRRMKRKKLNSRAK-VKS

>Ctel_Post2 [Capitella teleta]

---PKQRKKRKPYTRYQTMVLENEFINNSYITRQKRWEISCKLHLSERQIKVWFQNRRMKRKKLNERAK---S

>Esco_Post2

---TKGRKKRKPYTRYQTMVLENEFLNSSYITRQKRWEISCKLQLTERQIKVWFQNRRMKRKKLNERAK-ARL

>Obim_Post2

---TKGRKKRKPYTRYQTMVLENEFLNSSYITRQKRWEISCKLQLTERQIKVWFQNRRMKRKKLNERAK-ARL

>Pau_Post2

-----RGKKRKPYTRYQNMVLENEFLSSSYITRQKRWEISCKLHLTERQIKVWFQNRRMKRKKINERAK-ALF

>Pha_Post2

---IRTRKKRKPYTRYQNMVLENEFLGSSYITRQKRWEISCKLHLTERQIKVWFQNRRMKRKKINERAK-ALF

>Lana_Post2

---AKQRKKRKPYTRYQTMVLENEFLNNAYITRQKRWEISCKLHLSERQIKVWFQNRRMKRKKLNERAK-ALF

>Nano_Post2

---IRTRKKRKPYTRYQTMVLENEFLNSAYITRQKRWEISCKLHLSERQIKVWFQNRRMKRKKLNERAK-ALF

>Ttra_Post2

---FRSRKKRKPYTRYQNMLLENEFIANSYITRQKRWEISCKLQLTERQIKVWFQNRRMKRKKLTDRAK-SLF

>Ctur_Post2 [Crisularia turrita]

---SSSRKKRKPYTRYQTMVLETEFINNSYITRQKRWEISCRLRLTERQIKVWFQNRRMKRKKLNDRAKNAQL

>Fenf_Hox3 [Flaccisagitta enflata]

-------------------------HFNRYLCRPRRIEMAALLNLSERQIKI---------------------

>Fenf_PostA [Flaccisagitta enflata]

------RKKRKPYTKHQTFILEQEYLMSTYITRQRRLELARNLSLTERQIKIWFQNRRMKTKKLRERNKVSVG

>Fenf_PostB [Flaccisagitta enflata]

TSGGKCRTKRKPYEKWVTYLLEEEYLSNTYITKQKRYELSYRTSLTERQIKIWFQNRRMKSKKLRERSTSGAT

>Ctel_Cdx [Capitella teleta]

GKTRTKDKYRIVYSEYQKVELEKEYLYSKYITIQRKAELSRSIGLSERQIKIWFQNRRAKERKQKRKMEEALS

>Ctel_Post1 [Capitella teleta]

---VNPKKKRKPYSKPQVSALENEYSTSTYITKARRKEVARELDLTERQIKIWYQNRRIKEKKIATKRAKVQS

>Nano_Evx evx [Novocrania anomala]

QSDDNIRRYRTAFTREQISRLEKEFYKENYVSRPRRCELAASLNLPESTIKVWFQNRRMKDKRQRMAVAWPYG

>Lana_Evx [Lingula anatina]

PDDGGVRRYRTAFTREQIGRLEKEFYKENYVSRPRRCELAAALNLPESTIKVWFQNRRMKDKRQRMALAWPYG

>Ttra_Evx evx [Terebratalia transversa]

TDENGIRRYRTAFSREQIDKLEKEYSKENYVSRPRRCELASSLNLPESTIKVWFQNRRMKDKRQRMAVAWPFA

>Ctel_Evx1 even-skipped 1 [Capitella teleta]

VDDNGIRRYRTAFTREQLGRLEREFLKENYVSRPRRCELAASLNLPESTIKVWFQNRRMKDKRQRLAMAWPYG

>Ctel_Evx2 even-skipped 2 [Capitella teleta]

VDDNGIRRYRTAFTREQLGRLEREFLKENYVSRPRRCELAASLNLPESTIKVWFQNRRMKDKRQRLAMAWPYG

>Cgig_Evx [Crassostrea gigas]

AQERDIRRYRTAFTKEQLNRLEKEFLKENYVSRPKRCELAAQLNLSESTIKVWFQNRRMKDKRQRMALTWPYG

**Alignment of Fox sequences used for phylogenetic analysis (Fig. 2) in FASTA format:**

>SccFox1

KPPQSYASMITQAILSTPEGSISLADIYKFISDNYAFYRF-----SQMAW

QNSVRHNLSLNKAFEKVPKR----AGQ-QGKGMNWKIS

>HsFoxN1

KPIYSYSILIFMALKNSKTGSLPVSEIYNFMTEHFPYFKT-----APDGW

KNSVRHNLSLNKCFEKVENK----SGSSSRKGCLWALN

>HsFoxN4

KPIYSYSCLIAMALKNSKTGSLPVSEIYSFMKEHFPYFKT-----APDGW

KNSVRHNLSLNKCFEKVENK----MSGSSRKGCLWALN

>SkFoxN14

KPAYSYSCLIAMALKNSKTGCLPVSEIYHFMCENFPYFKT-----APDGW

KNSVRHNLSLNKCFEKIEKP---PGGGSTRKGCLWALN

>SpFoxN14

KPAYSYSCLITMSLKNSQNGCLPVSEIYQFMCENFPYFKT-----APDGW

KNSVRHNLSLNKCFAKIEKPQVNNGNGSARKGCLWAMN

>BfFoxN14a

KPAYSYSCLIAMALKNSKTGCLPVSEIYNFMCDNFPYFKT-----APDGW

KNSVRHNLSLNKCFEKVEKS----TGGTSKKGCLWTLN

>DmFoxN14

KPAYSYSCLIALALKNSRAGSLPVSEIYSFLCQHFPYFEN-----APSGW

KNSVRHNLSLNKCFEKIERP----ATNGNRKGCRWAMN

>BfFoxN14b

KPPYSYSCLIALAMMKSRTGNMPVTEIYSFIREHYPYFKT-----APDGW

KNSVRHNLSLNKCFLKIEPS----QADPNRKGCLWALH

>BfFoxM

RPPYSYMALIQFAINSSPARRMTLKEIYTWIEGRFPYFKT-----AKQGW

KNSIRHNLSLHDIFIRETTK----C-ESGRALSYWTLC

>CiFoxM

RPPYSYMALIQFAINSSSTGKMTLRQIYQWIEEKFPYFKT-----AKPGW

KNSIRHNLSLHDIFVRQVT--------TGSKASYWTLR

>HsFoxM1

RPPYSYMAMIQFAINSTERKRMTLKDIYTWIEDHFPYFKH----IAKPGW

KNSIRHNLSLHDMFVRETS--------ANGKVSFWTIH

>SpFoxM

RPPYSYSSLIQFAISSAPEGKLTLRDVYFWIETHFPYFRT-----AKLGW

KNSIRHNLSLHKIFVREAP-------SGPGQPAFWTLR

>SkFoxM

RPSYSYMSMIQFAINSKPDKKMTLQEIYHWVESTFPYFQS-----AKPGW

KNSIRHNLSLHDVFVREKP-------EVNGKSSFWKLK

>HsFoxO1

WGNLSYADLITKAIESSAEKRLTLSQIYEWMVKSVPYFKDKGDSNSSAGW

KNSIRHNLSLHSKFIRVQN-------EGTGKSSWWMLN

>BfFoxO

WGNLSYADLITKAIQSSPEGRLTLSQIYDWMVRCVPYFRDKGDSNSSAGW

KNSIRHNLSLHSRFIRVQN-------EGTGKSSWWMLN

>SkFoxO

WGNLSYADLITKAIESAPDKRLTLSQIYEWMVKSVPYFKDKGDSNSSAGW

KNSIRHNLSLHSRFVRVQN-------EGTGKSSWWMIN

>SpFoxO

WGNLSYADLITKAIQSAPDQRLTLSQIYDWMVKNVPFFKDKGDSNSSAGW

KNSIRHNLSLHSRFVRVQN-------EGTGKSSWGXST

>HsFoxO3

WGNLSYADLITRAIESSPDKRLTLSQIYEWMVRCVPYFKDKGDSNSSAGW

KNSIRHNLSLHSRFMRVQN-------EGTGKSSWWIIN

>DmFoxO

WGNLSYADLITHAIGSATDKRLTLSQIYEWMVQNVPYFKDKGDSNSSAGW

KNSIRHNLSLHNRFMRVQN-------EGTGKSSWWMLN

>CiFoxO

WGNMSYADLITQGIESSPDKRLTLAQIYDWMVKNVPYFKDKGDSNSSAGW

KNSIRHNLSLHSKFKRIQN-------EGTGKSSWWIIN

>NvFoxO_2

WGNYSYADLITQAIQSSPEKRLTLSQIYDWMVNSVPYFRDKGDSNSSAGW

KNSIRHNLSLHSKFVRVQN-------EGNGKSSWWVLN

>HsFoxO4

WGNQSYAELISQAIESAPEKRLTLAQIYEWMVRTVPYFKDKGDSNSSAGW

KNSIRHNLSLHSKFIKVHN-------EATGKSSWWMLN

>ChFoxO

WGNLSYADLITRAIQSSPEQRLTLSQIYEWMVRNIPYFKDKGDSTSSAGW

KNSIRHNLSLHSRFMRVQN-------DNNGKSSYWVIN

>NvFoxO_1

WGSESYSEMISKAIMSCPSQEATLHTIYEWIVNNVSYFADKADYPSTHGW

KNSIRHNLSLHSKFVRVQN-------EGNGKSSWWVLN

>CeDAF16

WGEESYSDIIAKALESAPDGRLKLNEIYQWFSDNIPYFGERSSPEEAAGW

KNSIRHNLSLHSRFMRIQN-------EGAGKSSWWVIN

>HsFoxF1

KPPYSYIALIVMAIQSSPTKRLTLSEIYQFLQSRFPFFRG-----SYQGW

KNSVRHNLSLNECFIKLPKG----LGRP-GKGHYWTID

>BfFoxF

KPPYSYIALIVMAIQSSATKRLTLSEIYQFLQQRFPFFRG-----PYQGW

KNSVRHNLSLNECFIKLPKG----LGRP-GKGHYWTID

>HsFoxF2

KPPYSYIALIVMAIQSSPSKRLTLSEIYQFLQARFPFFRG-----AYQGW

KNSVRHNLSLNECFIKLPKG----LGRP-GKGHYWTID

>SkFoxF

KPPYSYIALIVMAIQSSPTKRLTLSEIYQFLMNRFPFFRG-----PYQGW

KNSVRHNLSLNECFIKLPKG----LGRP-GKGHYWTID

>SpFoxF

KPPYSYIALIVMAIQSSPAKRLTLSEIYQFLMQRFPFFRG-----PYQGW

KNSVRHNLSLNECFIKLPKG----LGRP-GKGHYWTID

>CiFoxF

KPPYSYIALIVMAIQSSPAKKLTLSEIYNFLQTRFEFFRG-----AYQGW

KNSVRHNLSLNECFIKLPKG----LGRP-GKGHYWTID

>DmFoxF

KPALSYINMIGHAIKESPTGKLTLSEIYAYLQKSYEFFRG-----PYVGW

KNSVRHNLSLNECFKKLPKG----MGKP-GKGNYWTID

>CeLet381

--PFSYIALIAMAISKRPDKKATLAEIYSYLQENFEFFRG-----EYAGW

RNSIRHNLSLNECFVKLPKD----TGES-GKGHKWTIS

>HsFoxH1

KPPYTYLAMIALVIQAAPSRRLKLAQIIRQVQAVFPFFRE-----DYEGW

KDSIRHNLSSNRCFRKVPKD----PAKPQAKGNFWAVD

>BfFoxH

KPPYSYLALVVMAIQNAPEKKLPLKEIHEALKKMYPFFRG-----DYTGW

KDSVRHNLSTYKCFYKVPKD----PSRPFAKGNYWAVY

>BfFox1

KPPVSYTAMIAAVIQESPEKKLTLLQIVDELKKRYSFFNG-----DYKGW

KNSVRHNLSLNKCFVKVPRD----ADRPFGKDNFWTVD

>CiFoxH

KPPYSYVSLITLSILSSPEKKLRLSQILKRISEMFPFFNG-----SYQGW

RDSVRHNLSQNECFVKVLKN----PYRPTAKGNYWTVN

>SpFoxQ1

KPPFSYIALIAMAIRDSGHGKLTLAEINEYLMKKFPFFRG-----SYTGW

RNSVRHNLSLNECFRKILRD----PSRPWGKDNYWTIN

>CiFoxQ1

KPPFSYIALIAMAIRDSANGKLTLAEINEYLMKKFPFFRG-----SYTGW

RNSVRHNLSLNECFQKILRD----PSRPWGKDNYWTIN

>BfFoxQ1

KPPFSYIALIAMAIRDSPNNRLTLAEINDYLMKKFEFFRG-----PYTGW

RNSVRHNLSLNECFTKVLRD----PSRPWGKDNYWTIN

>SkFoxQ1

KPPLSYIALIAAAIQESPTRKLTLAEINDYLMKKYPFFRG-----SYTGW

RNSVRHNLSLNECFTKVLRD----PSRPWGKDNYWTIN

>HsFoxQ1

KPPYSYIALIAMAIRDSAGGRLTLAEINEYLMGKFPFFRG-----SYTGW

RNSVRHNLSLNDCFVKVLRD----PSRPWGKDNYWMLN

>HsFoxA1

KPPYSYISLITMAIQQAPSKMLTLSEIYQWIMDLFPYYRQ-----NQQRW

QNSIRHSLSFNDCFVKVARS----PDK-PGKGSYWTLH

>HsFoxA3

KPPYSYISLITMAIQQAPGKMLTLSEIYQWIMDLFPYYRE-----NQQRW

QNSIRHSLSFNDCFVKVARS----PDK-PGKGSYWALH

>XlFoxA4

KPPYSYISLITMAIQQAPNKMMTLNEIYQWIIDLFPYYRQ-----NQQRW

QNSIRHSLSFNDCFVKVPRS----PEK-PGKGSYWTLH

>HsFoxA2

KPPYSYISLITMAIQQSPNKMLTLSEIYQWIMDLFPFYRQ-----NQQRW

QNSIRHSLSFNDCFLKVPRS----PDK-PGKGSFWTLH

>NvFoxA

KPPYSYISLITMAIQQSPNKMLTLSEIYQFIMDLFPYYRQ-----NQQRW

QNSIRHSLSFNDCFVKVPRS----PDR-PGKGSYWTLH

>CiFoxA

KPPYSYISLITMALQSSPNKMMTLSEIYNWIMDLFPFYRQ-----NQQRW

QNSIRHSLSFNDCFVKVPRS----ADK-PGKGSYWSLH

>CeFoxA

KPPYSYISLITMAIQKSNSRQLTLSEIYNWIMDLFPYYQN-----NQQRW

QNSIRHSLSFNDCFVKVARS----PDK-PGKGSFWTLH

>SkFoxA

KPPYSYISLITMAIQSSPNKMVTLSDIYQFIMDLFPFYRQ-----NQQRW

QNSIRHSLSFNDCFLKVPRT----PDR-PGKGSFWTLH

>SpFoxA

KPPYSYISLITMAIQQSPQKMVTLSDIYQFIMDLFPFYRQ-----NQQRW

QNSIRHSLSFNDCFVKVPRT----PDR-PGKGSFWTLH

>BfFoxA_a

KPPYSYISLITMSIQSSPNKMVTLAEIYQFIMDLFPYYRQ-----NQQRW

QNSIRHSLSFNDCFVKVPRT----PDR-PGKGSYWTLH

>BfFoxA_b

KPPYSYIALITMAVQSSPNKMVTLSEIYQFIMDLFPFYRQ-----NQQRW

QNSIRHSLSFNDCFVKVQRT----PDR-PGKGSYWTLH

>DmFoxA

KPPYSYISLITMAIQNNPTRMLTLSEIYQFIMDLFPFYRQ-----NQQRW

QNSIRHSLSFNDCFVKIPRT----PDK-PGKGSFWTLH

>HvFoxA

KPPYSYISLITMSIQNTPGKAVTLSEIYQFIMDHFPYYRQ-----NQQRW

QNSIRHSLSFNDCFIKVPRS----PDK-PGKGSFWTLH

>HmFoxA

KPPYSYISLITMSIQNTPGKAVTLSEIYQFIMDHFPYYRQ-----NQQRW

QNSIRHSLSFNDCFIKVPRS----PDK-PGKGSFWTLH

>HsFoxB1

KPPYSYISLTAMAIQSSPEKMLPLSEIYKFIMDRFPYYRE-----NTQRW

QNSLRHNLSFNDCFIKIPRR----PDQ-PGKGSFWALH

>BfFoxB

KPPYSYISLTAMAIQSSGEKMLPLSDIYKFIMDRFPFYRQ-----NTQRW

QNSLRHNLSFNDCFIKIPRR----PDQ-PGKGSFWALH

>HsFoxB2

KPPYSYISLTAMAIQHSAEKMLPLSDIYKFIMERFPYYRE-----HTQRW

QNSLRHNLSFNDCFIKIPRR----PDQ-PGKGSFWALH

>SkFoxB

KPPYSYIALTAMAIQSSTEKMLPLSDIYKFIMDRFPFYRK-----NTQRW

QNSLRHNLSFNDCFIKIPRR----PDR-PGKGSYWALH

>SpFoxB

KPPYSYISLTAMAIQSSQEKMLPLSDIYKFIMDRFPYYRK-----NTQRW

QNSLRHNLSFNDCFLKIPRR----PDR-PGKGSYWALH

>DmFoxB

KPPYSYISLTAMAIWSSPEKMLPLSDIYKFITDRFPYYRK-----NTQRW

QNSLRHNLSFNDCFIKVPRR----PDR-PGKGAYWALH

>CeFoxB

KPPYSYIWLTYMAIQDSDDKMLPLTEIYKYIMDRFPFYRK-----NTQRW

QNSLRHNLSFNDCFIKIPRR----ADR-PGKGSYWAVH

>CiFoxB

KPPYSYIALTAMAIQSSPNKMMSLSEIYRYIMDRFPFYRN-----NTQRW

QNSLRHNLSFNDCFVKVPRR----GDQ-PGKGSLWSLH

>NvFoxB

KPPYSYISLTAMAIQSSPQKMLSLSEIYQFIMDHFPFYRD-----NTQRW

QNSLRHNLSFNDCFVKIPRR----PDQ-PGKGSLWALH

>ChFoxB

KPPYSYVALCAMAIHSSPAKMMTLSQIYKFIMDNFPFYRK-----NSTRW

QNSLRHNLSFNDCFVKVSKT----SEH-GGKGNYWTLH

>HvFkh3

KPPYSYVALCAMAIHSSPYQMMTLSDIYKYIMNKFPFYRK-----NNKKW

QNSLRHNLSFNDCFVKISKT----SKP-GGKGNYWTMH

>SkFoxAB

KPPYSYIALIAMSLENAQDGMLTLNEVYEFIMNKFPYFRE-----NQQRW

QNSIRHNLSLNDCFVKIPRA----PGR-AGKGNYWALH

>SpFoxAB

KPPYSYIALITMALEGSKDGMMTLNEVYQFIMDKFPYFRE-----NQQRW

QNSIRHNLSLNDCFIKVPRA----PGR-PGKGNYWALH

>NvFox3

KPPFSYIALITMSIEASPYRMRTLNEIYEFIMTRFPYFRK-----NQQKW

QNSIRHNLSLNDCFVKVPRS----FGK-PGKGNYWTLH

>BfFoxAB

KPPYSYIALIVMALRSSPLGALPLTGIYEFIERTFPYFRR-----NKRRW

QNSIRHNLSLNDCFVKIPRS----CEQ-PGKGGLWALH

>HsFoxE1

KPPYSYIALIAMAIAHAPERRLTLGGIYKFITERFPFYRD-----NPKKW

QNSIRHNLTLNDCFLKIPRE----AGR-PGKGNYWALD

>HsFoxE3

KPPYSYIALIAMALAHAPGRRLTLAAIYRFITERFAFYRD-----SPRKW

QNSIRHNLTLNDCFVKVPRE----PGN-PGKGNYWTLD

>XlFoxE4

KPPYSYIALIAMAIANSPERKLTLGGIYKFIMERFPFYRE-----NSKKW

QNSIRHNLTLNDCFVKIPRE----PGH-PGKGNYWTLD

>SkFoxE

KPPYSYIALIAMSIANSPERKLTLGGIYKFIMDRFPFYRD-----NSKKW

QNSIRHNLTLNDCFVKLPRE----PGR-PGKGHYWTLD

>BfFoxEa

KPPYSYIALISMAIANSPERKLTLGGIYKFIMDRFPYYRD-----RDKKW

QNSIRHNLTLNDCFVKIPRE----PGR-PGKGNYWTLD

>CiFoxE

KPPYSYIALISMAIASSPERKLTLGHIYKFIMERFPFYRE-----QNKKW

QNSIRHNLTLNDCFIKLPRE----PGK-PGKGNYWTLD

>NvFoxE

KPPYSYIALICMAITSSPQRQLTLSEIYDFISQRFPFYQT-----CSIKW

KNSIRHNLTLNDCFIKLPRE----PNR-PGKGNYWTID

>BfFoxEb

KPPYSYVALITMAIVNSPERKTTLAGIYKFIMDHFPYYRE-----ADKKW

QNSIRHNLTLNDCFVKLARH----PNR-PGKGSLWALD

>BfFoxEc

KPPYSYVALITMAIVNSPERKTTLAGIYKFIMDHFPYYRE-----ADKKW

QNSIRHNLTLNDCFVKLARH----PNR-PGKGSLWALD

>HsFoxD1

KPPYSYIALITMAILQSPKKRLTLSEICEFISGRFPYYRE-----KFPAW

QNSIRHNLSLNDCFVKIPRE----PGN-PGKGNYWTLD

>HsFoxD2

KPPYSYIALITMAILQSPKKRLTLSEICEFISGRFPYYRE-----KFPAW

QNSIRHNLSLNDCFVKIPRE----PGN-PGKGNYWTLD

>HsFoxD3

KPPYSYIALITMAILQSPQKKLTLSGICEFISNRFPYYRE-----KFPAW

QNSIRHNLSLNDCFVKIPRE----PGN-PGKGNYWTLD

>SpFoxD

KPPYSYIALITMSILQSPQKRLTLSGICEFIMNRFPYYRE-----KFPVW

QNSIRHNLSLNDCFVKIPRE----PGN-PGKGNYWTLD

>SkFoxD

KPPYSYIALITMAVLQSPQKRLTLSGICEFIMNRFPYYRE-----RFPVW

QNSIRHNLSLNDCFVKIPRE----PGN-PGKGNYWTLD

>BfFoxD

KPPYSYIALITMSILQSPQKKLTLSQICEFIMNRFPYYRE-----RFPVW

QNSIRHNLSLNDCFVKIPRE----PGN-PGKGNYWTLD

>CiFoxD

KPPYSYIALITMSILQSPDKKLTLSGICDFIMNRFPYYKE-----KFPAW

QNSIRHNLSLNDCFVKIPRE----PGN-PGKGNYWTMD

>NvFoxD2

KPPYSYIALITMAILQSPQRKLTLSDICEFIKRRFPYYRE-----KFPSW

QNSIRHNLSLNDCFVKMPRE----PGN-PGKGNYWTLD

>HsFoxD4

KPPSSYIALITMAILQSPHKRLTLSGICAFISDRFPYYRR-----KFPAW

QNSIRHNLSLNDCFVKIPRE----PGR-PGKGNYWSLD

>DmFoxD

KPPYSYIALITMAILQSPHKKLTLSGICDFIMSRFPYYKD-----KFPAW

QNSIRHNLSLNDCFIKVPRE----PGN-PGKGNFWTLD

>CeFoxD

KPPYSYIALIAMSILNSPEKKLTLSEICEFIINKFEYYKE-----KFPAW

QNSIRHNLSLNDCFVKVARG----PGN-PGKGNYWALD

>SdFoxD

KPPYSYIALIAMAISHSPNKMLTLGEICDYIIHQFTYYHK-----RWPAW

QNSIRHNLSLNDCFIKVPRE----YGS-SGKGNFWKLH

>NvFox6

KPPYSYIALIAMAIVEAPDKRRTLSEIVEFIKRRFSYYRD-----CIKGW

QNSIRHNLSLNDCFIKTWRD----PTN-PSKGHLWTLH

>ReFoxL1

KPAYSYIALIAMSIECAPHKRATLSEICQFIRDRFPYYQN-----CKQGW

ENSIRHNLSLNECFVKQPRE----QGR-PGKGHYWTLD

>SdFoxF

KPPYSYIALITLAIMSKAERKATLAEICQYIRETFSYYRE-----NKQGW

ENSIRHNLSLNQCFQKLPRE----QGK-PGKGHYWVID

>HsFoxC1

KPPYSYIALITMAIQNAPDKKITLNGIYQFIMDRFPFYRD-----NKQGW

QNSIRHNLSLNECFVKVPRD----DKK-PGKGSYWTLD

>HsFoxC2

KPPYSYIALITMAIQNAPEKKITLNGIYQFIMDRFPFYRE-----NKQGW

QNSIRHNLSLNECFVKVPRD----DKK-PGKGSYWTLD

>SkFoxC

KPPYSYIALIAMAIQNAPEKKVTLNGIYQFIMDRFPFYRE-----NKQGW

QNSIRHNLSLNDCFIKVPRD----DKK-PGKGSYWSLD

>BfFoxC

KPPYSYIALIAMAIQNAPDKKVTLNGIYQFIMDRFPYYRE-----NKQGW

QNSIRHNLSLNECFIKVPRD----DKK-PGKGSYWSLD

>SpFoxC

KPPYSYIALIAMAIMNASDKKITLNGIYQFIMDRFPFYRE-----NKQGW

QNSIRHNLSLNDCFIKIPRD----DKK-PGKGSYWSLD

>DmFoxC

KPPYSYIALIAMAIQNAADKKVTLNGIYQYIMERFPYYRD-----NKQGW

QNSIRHNLSLNECFVKVARD----DKK-PGKGSYWTLD

>CiFoxC

KPPYSYIALIAMAIQNAPDKKVTLNGIYQWIMERFPFYRE-----NKQGW

QNSIRHNLSLNECFVKIPRD----DKK-PGKGSYWTMD

>NvFoxC

KPPYSYIALIAMAIQSAPEKRITLSGIYSFIMDRFPYYR------NNQGW

QNSIRHNLSLNECFVKVPRD----DKK-PGKGSFWMLD

>HsFoxS1

KPPYSYIALIAMAIQSSPGQRATLSGIYRYIMGRFAFYRH-----NRPGW

QNSIRHNLSLNECFVKVPRD----DRK-PGKGSYWTLD

>DgFoxL1

KPPYSYIALIAMAIQNASDKKVTLNGIYQYIMERFPYYRD-----NKQGW

QNSIRHNLSLNECFVKVARD----DKK-PGKGSYWTLD

>SpFoxL1

KPPYSYIALIAMAIRNSSDKKVTLNGIYQFIMDRFPYYHD-----NKQGW

QNSIRHNLSLNDCFVKVARE----KGK-PGKGNYWTLA

>SkFoxL1

KPPYSYIALIAMAIRSAPDQKTTLNGIYQFIMERFPYYHD-----NKQGW

QNSIRHNLSLNDCFVKVPRE----KGK-PGKGNYWSLA

>BfFoxL1

KPPYSYIALIAMAIRSSPDQKITLNGIYQWIMDRFPYYHD-----NKQGW

QNSIRHNLSLNDCFVKDCEE----MFE---NGNYRRRK

>HsFoxL1

KPPYSYIALIAMAIQDAPEQRVTLNGIYQFIMDRFPFYHD-----NRQGW

QNSIRHNLSLNDCFVKVPRE----KGR-PGKGSYWTLD

>HsFoxI1

RPPYSYSALIAMAIHGAPDKRLTLSQIYQYVADNFPFYNK-----SKAGW

QNSIRHNLSLNDCFKKVPRD----EDD-PGKGNYWTLD

>DrFoxI3

RPPYSYSALIAMAIHGAPNRRVTLSQIYQYVADNFPFYNK-----SKASW

QNSIRHNLSLNDCFMKVPRD----DSD-PGKGNYWTLD

>HsFoxI2

RPPYSYSALIAMAIQSAPLRKLTLSQIYQYVAGNFPFYKR-----SKAGW

QNSIRHNLSLNDCFKKVPRD----EDD-PGKGNYWTLD

>BfFoxI

RPPYSYSALIAMAIQAAPEKKLTLSGIYQYVADNFPFYKK-----SKAGW

QNSIRHNLSLNDCFKKVPRD----EDD-PGKGNYWTLD

>CiFoxI

RPPYSYSALIAMAIQNSPEKKLTLSQIYQYVAENFPFYKK-----SRAGW

QNSIRHNLSLNDCFKKVARD----EDD-PGKGNYWSLD

>SkFoxI

RPPYSYSALIAMAIQSAGEKKITLSGIYKYVSDNFPFYKK-----SKAGW

QNSIRHNLSLNDCFKKVPRS----EDD-PGKGNYWMLD

>SpFoxI

RPPYSYSALIAMAIQSSPDHKITLSGIYRYVAENFPFYKR-----SKAGW

QNSIRHNLSLNDCFIKVPRA----DND-PGKGHYWTLD

>HsFoxL2

KPPYSYVALIAMAIRESAEKRLTLSGIYQYIIAKFPFYEK-----NKKGW

QNSIRHNLSLNECFIKVPRE----GGG-ERKGNYWTLD

>NvFoxL2

KPPYSYVALIAMAIRESPEKRLTLNGIYQYIISKFPYYEK-----NKKGW

QNSIRHNLSLNECFIKVPRE----GGG-ERKGNYWTLD

>BfFoxL2

KPPYSYVALIAMAIKESQEKRLTLSQIYNYIIQKFPYYEK-----NKKGW

QNSIRHNLSLNECFIKVPRE----GGG-ERKGNYWTLD

>SkFoxL2

KPPYSYVALIAMAIRESQEKRLTLSQIYDFIVNKFPFYEK-----NKKGW

QNSIRHNLSLNECFIKIPRE----GGG-ERKGNFWTLD

>CiFoxL2

KPPYSYVALIAMAIRDSNEKKLTLSGIYQYIVDKFPFYEK-----NRKGW

QNSIRHNLSLNECFVKVPRE----GGG-ERKGNFWMLD

>SpFoxL2

KPPFSYVALIAMAIKDSPERKLTLSQIYQYIINKFSYYEK-----NKKGW

QNSIRHNLSLNECFLKIARE----GGGGEKKGNYWTLD

>SdFoxL2

KPPYSYVALIAMSIAKSPDKRLTLSGIYQYIMDNFPYYAK-----NKKGW

QNSIRHNLSLNECFVKVPKE----GGD--RKGNYWTLD

>BfFoxG

KPPFSYNALIMMAIRQSPEKRLTLNGIYEFIMKNFPYYRE-----NKQGW

QNSIRHNLSLNKCFVKVPRH----YDD-PGKGNYWMLD

>HsFoxG1

KPPFSYNALIMMAMRQSPEKRLTLNGIYEFIMKNFPYYRE-----NKQGW

QNSIRHNLSLNKCFVKVPRH----YDD-PGKGNYWMLD

>SkFoxG

KPPFSYNALIMMAIRQSPEKRLTLNGIYEFIMKHFPYYRE-----NKQGW

QNSIRHNLSLNKCFVKVPRH----YDD-PGKGNYWMLD

>SpFoxG

KPPFSYNALIMMAIRSSPEKRLTLNGIYEYIMTNFPYYRE-----NKQGW

QNSIRHNLSLNKCFVKVPRH----YDD-PGKGNYWMLD

>DmSlp2

KPPYSYNALIMMAIRQSSEKRLTLNGIYEYIMTNHPYYRD-----NKQGW

QNSIRHNLSLNKCFVKVPRH----YDD-PGKGNYWMLD

>CeFkh2

KPPFSYNALIMMAIKDSPEKRLTLAGIYEYIVTNYPFYRD-----NKQGW

QNSIRHNLSLNKCFVKVPRN----FDD-PGKGNYWMLD

>CiFoxG

KPPYSYNALIMMAIKKSPRKRLTLSQIYQYITTTFPYYKE-----NKQAW

QNSIRHNLSSNKCFVKVPRH----YDD-PGKGNYWMLD

>DmSlp1

KPPYSYNALIMMAIQDSPEQRLTLNGIYQYLINRFPYFKA-----NKRGW

QNSIRHNLSLNKCFTKIPRS----YDD-PGKGNYWILD

>MlFoxG

KPLFSYNALIAMAISQSPLKKLTLSEIYDFIIETFPYYRD-----NKKGW

QNSIRHNLSLNKCFVKVPRH----YND-PGKGNYWMLN

>NvFox1

KPPYSYVALISMAIKQSPGRKITLNGIYHFITSAFPYYTQ-----NKRGW

QNSIRHNLSLNRCFVKVHRE----KAD-PGKGCYWTLD

>NvFox5

KPPYSYVALISMAIKQSKGQKITLSGIYQFIIENFPYYRL-----NKRGW

QNSIRHNLSLNKCFVKIPRE----RSD-PGKGCYWALD

>NvFoxD1

RPPYSYIALIAMAVQNSPEKRLTLDGICKFIRDRFPFYRE-----TYPSW

KICIRNNLSLNDCFIKTG-I----KSDEPLKGNYWTLD

>SdFox1

RPPFSYITLISMAVKNAPTKKLTLNEIYSYIMDHFPFYRE-----NRRGW

QNSIRHNLSLNECFVKVPRD----KDDPPGKGNYWTLA

>SkFoxJ1

KPPYSYATLICMAMKETKKNKITLSAIYKWIQDNFMYYKV-----AEPSW

QNSIRHNLSLNKCFTKVPRR----KDE-PGKGGFWKID

>BfFoxJ1

KPPYSYATLICMAMKETKKSKITLSDIYKWIKTNFKYYEM-----AEPSW

QNSIRHNLSLNKCFTKVPRS----KNE-PGKGGFWKID

>SpFoxJ1

KPPYSYSTLIWMAMKESKKHKITLSSIYKWITENFKYYQV-----ADPSW

QNSIRHNLSLNKCFQKVPRK----KDE-PGKGGFWRID

>HsFoxJ1

KPPYSYATLICMAMQASKATKITLSAIYKWITDNFCYFRH-----ADPTW

QNSIRHNLSLNKCFIKVPRE----KDE-PGKGGFWRID

>Hvfkh2

KPPYSYAALIIMAMKSKVCGKMTLSEIYKWIGDHFPFYKY-----AEPSW

QNSIRHNLSLNKCFTKIPRN----KGD-PGKGGYWTVI

>ReFoxJ

------TTIIYLAIRSSKNDKVTLGEIYQWIKDHFMYYRV-----AEPTW

QNSVRHNLSLN---------------------------

>SkFoxJ2

KPPYSYANLITFAINSSPKKKMTLSEIYQWICENFPFYRE-----AGNGW

KNSIRHNLSLNKCFLKVPRS----KDD-PGKGSYWAID

>SpFoxJ2

KPPYSYANLITFAINSSPKKKMTLSEIYQWICENFPYYRE-----AGNGW

KNSIRHNLSLNKCFMKVPRS----KDD-PGKGSYWAID

>BfFoxJ23

KPPYSYANLITFAINSSPKKKMTLSEIYQWICDNFPYYRD-----AGNGW

KNSIRHNLSLNKCFLKVPRS----KDD-PGKGSYWAID

>HsFoxJ3

KPPYSYASLITFAINSSPKKKMTLSEIYQWICDNFPYYRE-----AGSGW

KNSIRHNLSLNKCFLKVPRS----KDD-PGKGSYWAID

>CiFoxJ2

KPPYSYASLISLAINSSNEKKMTLSEIYQWICKTFPYYSG-----AGTGW

KNSIRHNLSLNKCFMKVPRA----KDD-PGKGSYWAID

>HsFoxJ2

KPRYSYATLITYAINSSPAKKMTLSEIYRWICDNFPYYKN-----AGIGW

KNSIRHNLSLNKCFRKVPRP----RDD-PGKGSYWTID

>HsFoxK1

KPPFSYAQLIVQAISSAQDRQLTLSGIYAHITKHYPYYRT-----ADKGW

QNSIRHNLSLNRYFIKVPRS----QEE-PGKGSFWRID

>BfFoxK

KPPYSYAQLIVQAITSANDKQLTLSGIYAHITKNYPYYRT-----ADKGW

QNSIRHNLSLNRYFIKVPRS----QEE-PGKGSFWRID

>HsFoxK2

KPPYSYAQLIVQAITMAPDKQLTLNGIYTHITKNYPYYRT-----ADKGW

QNSIRHNLSLNRYFIKVPRS----QEE-PGKGSFWRID

>SkFoxK

KPPYSYAQLIVQAIISAQDKQLTLSGIYCHIMKNYPYYRS-----ADKGW

QNSIRHNLSLNRYFIKVPRS----QEE-PGKGSFWRLD

>SpFoxK

KPPYSYAQLIVQAIMSAQDKQLTLSGIYSYITKTYPYYRT-----ADKGW

QNSIRHNLSLNRYFIKVPRS----QEE-PGKGSFWRLD

>CiFoxK

KPPYSYAQLIIQAISSAPHRQLTLSGIYAHITKNYPYYRT-----ADKGW

QNSIRHNLSLNRYFVKVPRS----QEE-SGKGSFWKVD

>DmFoxK

KPPYSYAQLIVQAISAAPDKQLTLSGIYSFIVKHYPYYRE-----TNKGW

QNSIRHNLSLNRYFIKVARS----QDE-PGKGSFWRID

>HmFoxK

KPPYSYAQLIVQAITSSADKQLTLNGIYQFIMKNYPYYRI-----NDKGW

--------------------------------------

>SccFox2

KPPHSYATMITQAILSSPEGVISLADIYKYISSNYAYYRF-----AKSGW

QNSIRHNLSLNKAFEKVPRR----PNE-PGKGMKWRIS

>DebFkh

KPPYSYATMITQAILSNDDGVMSLSEIYNWIASHYAYYKY-----SKTGW

QNSIRHNLSLNKAFEKVPRR----PNE-PGKGMKWQIS

>SchFox2

KPPYSYSVMIAQAILSSSECMMTLSNIYSWISTHYPYYRT-----TKSGW

QNSIRHNLSLNKAFRKVPRK----SGE-QGKGMKWSIV

>AspFkh2

KPPYSYATLIAQAIFSSEEEKLTLNSIYNWIMDKYAFYRH-----SQSGW

QNSIRHNLSLNKAFQKVPRR----TDE-PGKGMKWQIA

>DrFoxQ2

KPAQSYIALISMAILDSDEKKLLLCDIYQWIMDHYPYFKS-----KDKNW

RNSVRHNLSLNECFIKAGRS----DN---GKGHFWAIH

>ChFoxQ2a

KPTQSYIALIATAILKSKDKRLVLSDIYKYILDNYSYFQS-----QDKSW

RNSIRHNLSLNECFIKVGRS----E----GKGHYWAIH

>SkFoxQ2-2

KPTHSYIALISMAILSTSERKMLLSEIYKYIMNNFPYYRN-----KEKSW

RNSVRHNLSLNECFIKNGRS----YN---GKGNYWSIH

>NvFox4

KPAHSYIALIAMAILSNSSKKMILGDIYQYISDNFPYYRN-----KDKSW

RNSIRHNLSLNECFITAGRS-----E---GKGNYWAIH

>SkFoxQ2-1

KPTESYIALIAKAILSVREQKMLLCDIYQNIMDIYPFYRN-----NDKSW

RNSIRHNLSLNECFIKNGRS----ND---GRGNYWSIH

>SpFoxQ2

KPPHSYIALIAMAIINSQDKHLLLCDIYEYIMKRFPFFKD-----NERSW

RNSIRHNLSLNECFIKAGRS----GD---GRGHFWAIH

>BfFoxQ2a

KPRHSYIALIAMAIMSSKDKRLLLGDIYQWIMDNFPFYRN-----NERSW

RNSIRHNLSLNDCFIKAGRS----QD---GKGNYWAIH

>BfFoxQ2b

KPPLSYIALIAKAILGSPAKRLSLGSIYQYITDNYPYYQN-----RGQGW

RNSVRHNLSLNDCFIKAGRC----ED---GKGNYWAIH

>SkFoxQ2Dol

KPSHSYIGLIAMAILKSKDRKMVLSDIYQYILDNYPYFRA-----RGPGW

RNSIRHNLSLNDCFVKAGRS----AN---GKGHYWAIH

>DmFoxQ2

KPQHSYIGLIAMAILSSTDMKLVLSDIYQYILDNYPYFRS-----RGPGW

RNSIRHNLSLNDCFIKSGRS----A----GKGHYWAIH

>BfFoxQ2c

KPSHSYIGLIAMAIMSSKEKKLVLSDIYKYILDNYPYFRN-----RGPGW

RNSIRHNLSLNDCFVKMGRS----AN---GKGHFWAVH

>CeFkh10

KPQHSYIGLIAMAILSSPQKKMVLAEVYEWIMNEYPYFRS-----RGAGW

RNSIRHNLSLNDCFVKAGRA----A----GKGHYWAVH

>ChFoxQ2b

KPNHSYISLIANAILSSKEKRLVLSDIYQFVLDTQPYFRK-----AGQGW

RNSIRHNLSLNECFVKAGRS----P----GKGHFWAIN

>HmFoxQ2b

KPSHSYISLIANAILASPDKRLVLSDIYKYVLERYDYFKK-----KGSGW

RNSIRHNLSLNDCFIKAGRS----PN---GKGHYWAIN

>NvFox2

KPSQSYIGLIGKAIMSVPQKKLVLSDIYNYILTHYPYFRN-----KGAGW

RNSIRHNLSLNECFVKVGRS----S----GKGHFWAIN

>HmFoxQ2a

RNTPSYTAIIAQAILSSKEKKLPLGDVYEYIAENFPEFLK-----KGQGW

RNCVRHNLSLSECFVKAGRA----RN---GRGNYWGIH

>HmFoxQ2c

EKIASYTEMIAKAIFSGKGNMSTLQDIYEFLIENFPILKS-----RGKSW

KNSVRHTLSLNEWFVKIPRT----DN---GKSCYWSIH

>SpFoxY

LPGFTYAELITMAIQSSPSRMMTIVDIQQFFRDRFPCFRT-----SYKGW

HNSIRHNLSARECFYKVPIV----DKRHKCRTHYWMIN

>HsFoxP1

RPPFTYASLIRQAILESPEKQLTLNEIYNWFTRMFAYFRR-----NAATW

KNAVRHNLSLHKCFVRVENV----KGAV------WTVD

>BfFoxP

RPPFTYASLIRQAIIESPEKQLTLNEIYNWFTRTFAYFRR-----NAATW

KNAVRHNLSLHKCFVRVENV----KGAV------WTVD

>HsFoxP4

RPPFTYASLIRQAILETPDRQLTLNEIYNWFTRMFAYFRR-----NTATW

KNAVRHNLSLHKCFVRVENV----KGAV------WTVD

>SkFoxP

RPPFTYAALIRQSIIDSPDGQLTLNEIYNWFTRTFAYFRR-----NAATW

KNAVRHNLSLHKCFVRVENV----KGAV------WTVD

>SpFoxP

RPPFTYAALIRQGIIDAPDRQLTLNEIYNWFTRTFAYFRR-----NAATW

KNAVRHNLSLHKCFVRVENV----KGAV------WTVD

>HsFoxP2

RPPFTYATLIRQAIMESSDRQLTLNEIYSWFTRTFAYFRR-----NAATW

KNAVRHNLSLHKCFVRVENV----KGAV------WTVD

>DmFoxP

RPPFTYASLIRQAIIDSPDKQLTLNEIYNWFQNTFCYFRR-----NAATW

KNAVRHNLSLHKCFMRVENV----KGAV------WTVD

>CiFoxP

RPPFTYASLIRQAVLEAPDHQMTLNEIYNWFQKKFAYFRR-----NAPTW

KNAVRHNLSLHKCFVRVENV----KGAV------WTVD

>CeFkh7

RPPYTYASLIRQAIMESSDCQLTLNEIYTWFTETFAYFRR-----NAATW

KNAVRHNLSLHKCFQRVEQV----KGAV------WTVD

>SdFoxP

RPPFTYASLIRQAILESSDQCLTLCEIYAWFMKNFVYFRD-----NNPTW

KNAIRHNLSLHKCFVRVELS----RGAV------WTVD

>HsFoxP3

RPPFTYATLIRWAILEAPEKQRTLNEIYHWFTRMFAFFRN-----HPATW

KNAIRHNLSLHKCFVRVESE----KGAV------WTVD

>SpFoxX

RPPHSYVRIVIMSLLDCPGHEATIREIYDMITYKFPYYQE-----NRLHW

KNSVRHNLTVFSCFERVITE----EGSTSRGSNRWRLI

>HsFoxN3

KPPYSFSCLIFMAIEDSPTKRLPVKDIYNWILEHFPYFAN-----APTGW

KNSVRHNLSLNKCFKKVDKE----RSQSIGKGSLWCID

>BfFoxN23

KPPYSFSCLIFMAIEDSPSKRLPVKEIYNWILEHFPYFVN-----APTGW

KNSVRHNLSLNKCFKKVEKE----KGQSIGKGSLWMID

>SkFoxN23

KPPFSFSCLIFMAVEDSPNKRLPVKDIYQWILDHFPYFQN-----APTGW

KNSVRHNLSLNKCFKKVEKE----KGQTIGKGSLWCID

>SpFoxN23

KPPFSFSCLIFMSIEDCPLKRLPVKEIYRYIQDHFPYFRT-----APTGW

KNSVRHNLSLNKCFRKVDKI----KGQSLGKGSLWCVD

>HsFoxN2

KPPYSFSLLIYMAIEHSPNKCLPVKEIYSWILDHFPYFAT-----APTGW

KNSVRHNLSLNKCFQKVERS----HGKVNGKGSLWCVD

>CiFoxN

KPPYSFSCLIFMAVEDSVEKRLPVKEIYSWVCKHFPYFKT-----APSGW

KNSIRHNLSLNRCFKKAEIP----NKRKEVKGSLWCID

>DmFoxN23

KPPYSFSSLIFMAIEGSNEKALPVKEIYAWIVQHFPYFKT-----APNGW

KNSVRHNLSLNKSFVKVEKA----PNMG--KGSLWRVE

>NvFoxNx

KPPYSFSSLIFMAIEESPNKRLPVKDIYNWIMDHFPYFRD-----ARLGW

KNSVRHNLSLNKCFKKVDKD----KGQVRRSFDMSLID

>HsFoxR1

RPPLNYFHLIALALRNSSPCGLNVQQIYSFTRKHFPFFRT-----APEGW

KNTVRHNLCFRDSFEKVPVSMQ-GGASTRPRSCLWKLT

>HsFoxR2

RPPLNCSHLIALALRNNPHCGLSVQEIYNFTRQHFPFFWT-----APDGW

KSTIHYNLCFLDSFEKVPDSLK-DEDNARPRSCLWKLT

>PhFoxGa

KPPFSYNALIMMAIRSSPEKRLTLNGIYEFIMKNFPYYRE-----NKQGW

QNSIRHNLSLNKCFVKVPRHY-----DDPGKGNYWMLD

>PhFoxGb

KPAYSYNALIMMAITHQADKKVTLSGIYEYITTNFPYYKD-----NKHGW

QNSIRHNLSLNKCFVKVPRRY-----DDPGKGHYWTLD

>PhFoxQ2a

KPSHSYIGLIAMAILSSPEKKLVLSDIYQWILDNYSYFRN-----RGPGW

RNSIRHNLSLNDCFVKSGRSAN-------GKGHYWAID

>PhFoxQ2b

KPFHSYIALISMAILSTKSRKMLLNDIYQYIMDSFPFYNT-----NEKAW

RNSIRHNLSLNECFVKCGRADN-------GKGHYWSIQ

**Alignment of Pax sequences used for phylogenetic analysis (Fig. 2) in FASTA format:**

>NanoPax4_6

SGVNQLGGVFVNGRPLPDSTRQRIVELAHSGARPCDISRILQVSNGCVSKILGRYYETGS

IRPRAIGGSKPR-VATPEVVTKIAVYKRECPSIFAWEIRDRLLSDGICTQDNIPSVSSIN

RVLRNLASDNQKP-AMYDKLGMQNGLAWPRPNYNPPPPPSQT--SVGD-VKKEESNA--Q

TNESDEQMRIRLKRKLQRNRTSFTNAQIESLEKEFERTHYPDVFARERLAQKIDLPEARI

QVFSNRRAKWRREEKLRNQRRDAASGVPINSSFPNSMYHPSIH--QPVASMADSY-----

-SMPTVPNYSLSNN---LSGSGYSRPSPYNHTSSM-----------GSSTGLISPGVSVP

VQVPGGSGAMASQ----------

>TtraPax4_6

SGVNQLGGVFVNGRPLPDSTRQRIVELAHSGARPCDISRILQVSNGCVSKILGRYYETGS

IRPRAIGGSKPR-VATPEVVQKIAHYKRECPSIFAWEIRDRLLQETVCSQENIPSVSSIN

RVLRNLTTDNQKG-AMYDKLGLLNGQPWPRHNSGSNQYNLTP--SPTP-AAMETKKETPQ

NGESDEQMRMRLKRKLQRNRTSFTNSQIEALEKEFERTHYPDVFARERLAQKIDLPEARI

QVFSNRRAKWRREEKLRNQRREAANGTPINSSFPNTWHPAIHG--ATLSESQTPY-----

-SSLPMPSYSLANNLDYISGSSYSRPPCNAVNNIS-------------STGLISPGVSVP

VQVPGGPDSMAAQ----------

>PharPax4_6

SGVNQLGGVFVNGRPLPDSTRQRIVELAHSGARPCDISRILQVSNGCVSKILGRYYETGS

IRPRAIGGSKPR-VATPEVVAKIAVYKRECPSIFAWEIRDRLLSEGCCSQDTIPSVSSIN

RVLRDLASENQKQGAMYDKLGLLNGQGWPPRNGGASQFSSQP--PLTPDIKKEGSNSEGN

NSNEGDDMRLRLKRKLQRNRTSFTNAQVEALEKEFERTHYPDVFARERLAQKIDLPEARI

QVFSNRRAKWRREEKLRNQRRDAASNPPINSSYN---MYSSIH--QPMVSMPESY-----

-SIPPLSNYSMSSA--IPDYSNYSRPSPGTVPS-Q-----------GSSTGLISPGVSVP

VQVPGGVASMSAG----------

>AentPax4_6

SGVNQLGGVFVNGRPLPDSTRQKIVELAHSGARPCDISRILQVSNGCVSKILGRYYETGS

IRPRAIGGSKPR-VATNDVVNKIAVYKRECPSIFAWEIRDRLLSDAVCNQENIPSVSSIN

RVLRNLANDNQK-GTMYDKLGILNGQAWPR-NGAPAPYHTPTPHSTPPVAKKERTEETPQ

NGETDEQMRMRLKRKLQRNRTSFTAEQIESLEKEFERTHYPDVFARERLAQKIDLPEARI

QVFSNRRAKWRREEKLRSQRRDSGNGSPLNGGFSNGMYPSLHQPIATMADTYSSP-----

-SIPPMSSYSLSNN--LSEYGNYSRTSCNAANMQSNGGSSGECSVYNSITGLISPGVSVP

IQVPGGAASMTSQ----------

>MmusPax6

SGVNQLGGVFVNGRPLPDSTRQKIVELAHSGARPCDISRILQVSNGCVSKILGRYYETGS

IRPRAIGGSKPR-VATPEVVSKIAQYKRECPSIFAWEIRDRLLSEGVCTNDNIPSVSSIN

RVLRNLASEKQ-QDGMYDKLRMLNGQTGSW--TRPGWYPGTSVPGQPTQDGCQQQESSNG

EDSDEAQMRLQLKRKLQRNRTSFTQEQIEALEKEFERTHYPDVFARERLAAKIDLPEARI

QVFSNRRAKWRREEKLRNQRRQASNTPPISSSFSTSVYQPIPQPTTPVSSFTSGSLTNTY

SALPPMPSFTMANN---PSVNHMQTHMNSQPMGTS---------------GLISPGVSVP

VQVPGSEMS---Q----------

>SkowPax4_6

SGVNQLGGVFVNGRPLPDSTRQKIVELAHSGARPCDISRILQVSNGCVSKILGRYYETGS

IRPRAIGGSKPR-VATPPVVGKIAQFKRECPSIFAWEIRDRLLQEQVCTQDNIPSVSSIN

RVLRTLAAEKNGPEPVFDKLRLLNGQTWPRTGTNGTTPTNTSSTEGLVCSKKDIDATDAS

GDDDEAQARLRLKRKLQRNRTSFTQYQIETLEKEFERTHYPDVFARERLAQKIDLPEARI

QVFSNRRAKWRREEKLRNQRRQMSSAPPINSSFTNSVYQPIPQPTTPMVPRTADS----Y

SALPPVPSFSMAT----ARSYSNSTHPIGQPPIQSTQTSGGHGGYGGKYLGLISPGVSVP

VQVPASASSHTSQ----------

>MmusPax4

SSVNQLGGLFVNGRPLPLDTRQQIVQLAIRGMRPCDISRSLKVSNGCVSKILGRYYRTGV

LEPKCIGGSKPR-LATPAVVARIAQLKDEYPALFAWEIQHQLCTEGLCTQDKAPSVSSIN

RVLRALQEDQS----------------------------------------LHWTQVLPS

PHSNCGAPRGPHPGTSHRNRTIFSPGQAEALEKEFQRGQYPDSVARGKLAAATSLPEDTV

RVFSNRRAKWRRQEKLKWEAQLPGASQNSPGIISAQQSPGSVPSAALPVLEPLSPTAPGR

CSSDTSSQAYLQPY----------------------------------------------

--LTTHPGQVPST----------

>MmusPax3

GRVNQLGGVFINGRPLPNHIRHKIVEMAHHGIRPCVISRQLRVSHGCVSKILCRYQETGS

IRPGAIGGSKPKQVTTPDVEKKIEEYKRENPGMFSWEIRDKLLKDAVCDRNTVPSVSSIS

RILRSKFGKGE---------------------EEEADLERKEAEESEKKAKHSIDGQSDE

GSDIDSEPDLPLKRKQRRSRTTFTAEQLEELERAFERTHYPDIYTREELAQRAKLTEARV

QVFSNRRARWRKQAGANQLMAFNHLIPAMPTLPTYQLSETSYQPTSIPQAVSDPSLPPST

VHQSTIPSNADSSSSNPMNPTGLLTNHGGVPHQPQLTGGLEPTTTVSASCSQRLEHMKNV

DSLPTSQTAGYSMDPVTGYQYGQ

>MmusPax7

GRVNQLGGVFINGRPLPNHIRHKIVEMAHHGIRPCVISRQLRVSHGCVSKILCRYQETGS

IRPGAIGGSKPRQVATPDVEKKIEEYKRENPGMFSWEIRDRLLKDGHCDRSTVPSVSSIS

RVLRIKFGKKE---------------------DDEEG--DKKEEDGEKKAKHSIDGRLDE

GSDVESEPDLPLKRKQRRSRTTFTAEQLEELEKAFERTHYPDIYTREELAQRTKLTEARV

QVFSNRRARWRKQAGANQLAAFNHLLPGMPTLPPYQLPDSTYPTTTISQ---DGGLPPST

MHQGGLAAAAAAADSNHMNP-SILSNPSAVPPQPQLHGGLDSASSISASCSQRADSIKPG

DSLPTSQTTGYSVDPVAGYQYSQ

>MmusPax2

GGVNQLGGVFVNGRPLPDVVRQRIVELAHQGVRPCDISRQLRVSHGCVSKILGRYYETGS

IKPGVIGGSKPK-VATPKVVDKIAEYKRQNPTMFAWEIRAQLLREGICDNDTVPSVSSIN

RIIRTKVQQPFHP--AGTGVSTASPPVSSASNNGEKRKREEVEVYTDPAHIRGGGGVSEG

SVPNGDSQSGVDSLRKHLRADTFTQQQLEALDRVFERPSYPDVFQASEH-----------

--EQG----------------------------------------------------NEY

S-LPALTPGLDEVKTGRDMTSPPTGQGSYPTSTLAFSGNPYSHPQYTAYNEAWRFSNP--

-----ALAAPR-SAPAARAAAYD

>MmusPax5

GGVNQLGGVFVNGRPLPDVVRQRIVELAHQGVRPCDISRQLRVSHGCVSKILGRYYETGS

IKPGVIGGSKPK-VATPKVVEKIAEYKRQNPTMFAWEIRDRLLAERVCDNDTVPSVSSIN

RIIRTKVQQPPNQ--SSHSISSVSTDSAGSS-SADTNKRKRDE-------------IQES

PVPNGHSLPGRDFLRKQMRGDLFTQQQLEVLDRVFERQHYSDIFTTTEP-----------

--EQT----------------------------------------------------TEY

SAMASLAGGLDDMKTGRDLASPPAGQGSYSAPTLTFSGSPYSHPQYSSYNDSWRFPNP--

-----GLPAARGAAPPAAATAYD

>MmusPax8

GGLNQLGGAFVNGRPLPEVVRQRIVDLAHQGVRPCDISRQLRVSHGCVSKILGRYYETGS

IRPGVIGGSKPK-VATPKVVEKIGDYKRQNPTMFAWEIRDRLLAEGVCDNDTVPSVSSIN

RIIRTKVQQPFNLCVATKSLSAVTPPESPQSDGNDNKRKMDDSD-------------QDS

CRLSIDSQSSSSGPRKHLRTDTFSQHHLEALECPFERQHYPEAYASPSH-----------

--EQGLYPLPLLNSALDDGKATLTSSNLSTHQTYPVVADPHSPFAIKQETPELSSSSSAF

LDLQQVGSGGPAGASGREMVGPTSGQGSYASSAIAYSGNAYSHTPYSSYSEAWRFPNS--

-----SLSTSRPSAPPTSATAFD

>Skow_Pax2B

GGVNQLGGVFVNGRPLPDVVRQRIVDLAHSGVRPCDISRQLRVSHGCVSKILGRYYETGS

IKPGVIGGSKPK-VATPKVVDKIADYKRQNPTMFAWEIRDRLLAEAVCDNENVPSVSSIN

RIVRNKMAEKATQNGSASS-SSPPTTITVTTDAGTEKRKRDSQ-------------HHES

QANGVISSPAMENDSRKRSRPTYTSEQIEALEKAFDRSHFPESASTGNYHDDMIVKNAYG

RDKNEQSANQGYQTLSPLAPVSMIQDVMVHSPPQQPIPSPYQTGSCIGGNPSPQSSGPTT

LTVLQPVASNNNMSQYSGQSRAAAAQSSYPSSTMSLPHASASYSAREPTDYSTQFSSVPY

THAQYSSSDAWSRYGSTGLLNRT

>AcriPax2_5_8

GGVNQLGGVFVNGRPLPDVVRQRIVELAHQGVRPCDISRQLRVSHGCVSKILGRYYETGS

IKPGVIGGSKPK-VATPKVVDAITMYKQENPTMFAWEIRDRLLSEAVCSQENVPSVSSIN

RIVRNKAADKAKSSPGLPSESTPSGELGPSRASPSESFKRKQDADITNGHAEVENSARTA

HSPTMGSQV---------------------------------------------------

------------------------------------------------------------

------------------------------------------------------------

-------AQPYPAQQYTTVTTAE

>MmusPax1

GEVNQLGGVFVNGRPLPNAIRLRIVELAQLGIRPCDISRQLRVSHGCVSKILARYNETGS

ILPGAIGGSKPR-VTTPNVVKHIRDYKQGDPGIFAWEIRDRLLADGVCDKYNVPSVSSIS

RILRNKIGSLAQPASKQPPPYQYPYPSPVSPTSIPRSWPSAHSVS---------------

------------------------------------------------------------

-------------------------------------------------------LGIRT

FMEQTGALAGSEGAVNGLEKPSASSLSAVGGFLPAQHGVYSAPAAGYLSPGPPWPPAQAP

PLTPHGALAAAMTFKHREGTDRK

>MmusPax9

GEVNQLGGVFVNGRPLPNAIRLRIVELAQLGIRPCDISRQLRVSHGCVSKILARYNETGS

ILPGAIGGSKPR-VTTPTVVKHIRTYKQRDPGIFAWEIRDRLLADGVCDKYNVPSVSSIS

RILRNKIGNLAQQSYKQHQPHIYSYPSPITAAALPRTWPSSHSVT---------------

------------------------------------------------------------

-------------------------------------------------------LGIRS

ITDQG---VSDSSPVNGLEKGAPNGLPAVSSFVSA-----------------PTPAQVSP

YMTYSAAGWQHAGSTPLSPHNCD

>SkowPax1_9

GEVNQLGGVFVNGRPLPNGIRLRIVELAQLGIRPCDISRQLRVSHGCVSKILARYNETGS

ILPGAIGGSKPR-VTTPTVVKHIKEYKRMDPGIFAWEIRDRLIADNVCDKYNVPSVSSIS

RILRNKVGNNTIQSGYDPVKLYPYPCGPGPANSVHDILGFRHAGM---------------

------------------------------------------------------------

-------------------------------------------------------QGPDH

QGPFAMYPDSTGHSDHQRSSMHLEGDTGKMSISPTGSFVPSTLTISPYHHPNPTYVPTYG

ATTAMSSSGNGFPCSSGTNLNSD

>TtraPax1_9

GEVNQLGGVFVNGRPLPNAIRLKIVEMAGVGVRPCDISRQLRVSHGCVSKILARYHETGS

ILPGTIGGSKPR-VTTPNVVKQIKEYKERDPGIFAWEIRDKLLTEGVCDKFNVPSVSSIS

RILRNKIGNPLQNSSPNRFFSSNIPQQNINQHQAHPVFPVSVSPS---------------

------------------------------------------------------------

-------------------------------------------------------VDTRT

TPTSRVSTSRCGWPTKFHSTSS--------------------------------------

-----------------------

>ObimPax1_9

---NQLGGVFVNGRPLPNAIRMRIVELAQLGVRPCDISRQLRVSHGCVSKILARYNETGS

ILPGAIGGSKPR-VTTPNVVRHIKIYKEKDPGIFAWEIRD--------------------

------------------------------------------------------------

------------------------------------------------------------

------------------------------------------------------------

------------------------------------------------------------

-----------------------
